# Improved Stability and Tunable Functionalization of Parallel β‐Sheets via Multicomponent N‐Alkylation of the Turn Moiety

**DOI:** 10.1002/anie.201912095

**Published:** 2019-12-04

**Authors:** Manuel G. Ricardo, Celia G. Moya, Carlos S. Pérez, Andrea Porzel, Ludger A. Wessjohann, Daniel G. Rivera

**Affiliations:** ^1^ Department of Bioorganic Chemistry Leibniz Institute of Plant Biochemistry Weinberg 3 06120 Halle/Saale Germany; ^2^ Faculty of Chemistry University of Havana 10400 Havana Cuba

**Keywords:** multicomponent reactions, peptides, secondary structures, solid-phase synthesis, β-sheets

## Abstract

In contrast to the myriad of methods available to produce α‐helices and antiparallel β‐sheets in synthetic peptides, just a few are known for the construction of stable, non‐cyclic parallel β‐sheets. Herein, we report an efficient on‐resin approach for the assembly of parallel β‐sheet peptides in which the N‐alkylated turn moiety enhances the stability and gives access to a variety of functionalizations without modifying the parallel strands. The key synthetic step of this strategy is the multicomponent construction of an N‐alkylated turn using the Ugi reaction on varied isocyano‐resins. This four‐component process assembles the orthogonally protected turn fragment and incorporates handles serving for labeling/conjugation purposes or for reducing peptide aggregation. NMR and circular dichroism analyses confirm the better‐structured and more stable parallel β‐sheets in the N‐alkylated peptides compared to the non‐functionalized variants.

## Graphical abstract

**Keep it folded**: An Ugi‐derived N‐alkylation suitably positioned at the turn moiety of a non‐cyclic parallel β‐sheet peptide enhances the folding propensity and thermal stability in aqueous solution. It also enables the installation of various functionalities, for example an extra cationic group to abolish aggregation, or a thiol for conjugation to a fluorescent label.

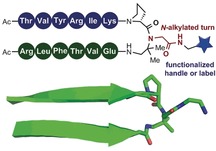

Many advances in modern peptide/protein science rest on the ability of synthetic chemists to ligate peptides, functionalize their side chains in a chemoselective manner and construct peptidomimetics capable of reproducing regular protein secondary structures in water. Whereas a variety of methods are available for producing water‐stable helical peptides^1^ and antiparallel β‐sheets,^2^ the synthesis of parallel β‐sheets that are stable in water is a notable endeavor.^3^ Understanding the stability and biological behavior of this subclass of secondary structure is crucial for modern science, but model parallel β‐sheets are more difficult to produce by synthetic means than the antiparallel variants, as they cannot be fully composed of α‐amino acids.^2^, ^3^ Problems associated with the inability to fold and the tendency to aggregate also make their study in water very challenging.^3^, ^4^, ^5^, ^6^ In this regard, many efforts have been devoted to producing non‐ and pseudo‐peptidic turns capable of properly aligning two parallel strands and promoting β‐sheet nucleation.^3^, ^4^, ^5^, ^6^ However, just a few non‐cyclic peptides are known to autonomously fold into a parallel β‐sheet in aqueous solution.^3^, 5b, 7 Besides the early contributions from Nowick^4^ and Kelly,^5^ Gellman's^3^, ^7^ group has provided the major advances by developing an effective pseudo‐peptidic turn.7a, 7b This group has also assessed the effect of strand length7c and number7d on the stability of non‐cyclic parallel β‐sheets in water.

As shown in Scheme 1, Gellman's approach^7^ comprises the utilization of a central turn moiety composed of d‐Pro and 1,2‐diamino‐1,1‐dimethylethane (DADME). Different variants of parallel β‐sheets have been produced using this turn,^8^ including examples that rely on backbone cyclization8a and interstrand disulfide cross‐linking8b to impose further macrocyclic constraints and improve stability. Considering the turn‐inducing capacity of amide N‐alkylation in short peptides,^9^ we hypothesized that the combination of d‐Pro with an N‐alkylated DADME moiety could serve as an effective template for assembling very stable parallel β‐sheets without the need of cyclization constraints. The rationale for this is the presence of an additional tertiary amide with a bulky N‐alkylation at the turn fragment, which should bend the peptide chain more easily and enforce the interstrand interactions to a greater degree.

**Scheme 1:**
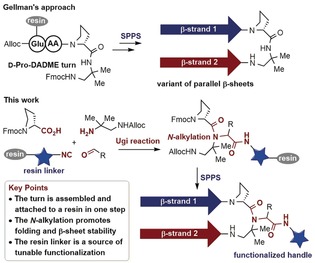
Comparison of state‐of‐the‐art with the novel multicomponent reaction (MCR) approach towards stable and functionalized parallel β‐sheets.

Herein, we introduce a multicomponent reaction (MCR) approach for the synthesis of water‐stable and functionalized parallel β‐sheets. Our strategy stands over key structural evidence previously validated by Gellman's group,^7^ but it provides a series of innovations in terms of synthetic efficiency, versatility, and β‐sheet stability, that make it very relevant for the peptide/protein community. As depicted in Scheme 1, we devised an approach relying on the Ugi‐multicomponent reaction^10^ to assemble the d‐Pro‐DADME turn moiety and simultaneously link this N‐alkylated fragment to the resin. In contrast to previous stepwise protocols, comprising the initial solution‐phase coupling of the d‐Pro‐DADME fragment to a short peptide followed by anchoring to the resin by a side chain,^7^ our strategy is very convergent and fully conducted on solid phase. In this sense, each Ugi‐component has a different purpose, i.e., while Fmoc‐d‐Pro and Alloc‐DADME serve as carboxylic acid and amine components, respectively, the isocyanide enables the attachment to the resin and introduces the functional linker. The choice of the carbonyl component is also relevant since the use of a cleavable aldehyde allows access to non‐alkylated β‐sheets.

Previously, MCRs have been used to produce conformationally constrained macrocyclic peptides^11^ featuring stable protein secondary structures, such as α‐helices^12^ and reverse turns.^13^ However, this chemistry has never been employed for the construction of non‐cyclic β‐sheets. In contrast to the clear conformational bias imposed by a macrocyclization, here we aim at addressing the effect of the Ugi‐derived N‐alkylation of a model turn moiety on the stability of non‐cyclic parallel β‐sheets. Evidence for the effectiveness of d‐Pro‐l‐Pro as a template of antiparallel β‐sheets suggests that two consecutive tertiary amides at the central turn fragment should confer a preorganization for folding antiparallel β‐hairpin conformations.^2^, ^3^ We wondered whether the turn connecting two parallel strands might follow the same tendency and benefit from an additional N‐alkylation of the artificial DADME residue. Previous reports of our group on the turn‐forming propensity of Ugi‐derived N‐alkylated peptide fragments shed light on this possibility.^14^

By focusing on an MCR approach, we also seek to provide a flexible protocol for producing parallel β‐sheets with varied types of functionalization at the turn moiety. As β‐sheet peptides are endowed with low solubility and tendency to aggregate, we envisioned that an additional functionalization at a position not affecting the parallel strands would be beneficial not only for the solubility but also for late‐stage derivatization purposes (for example, labeling). As depicted in Scheme 2, a key advantage of this approach is that the complete sequence can be carried out on resin, which needs to be initially functionalized with an isocyanide group.^15^ Four different commercially available amino resins, i.e., Rink‐MBHA, Gly‐Wang, ethylenediamine‐trityl, and 2‐aminoethyl‐polystyrene, were initially modified by a formylation/dehydration protocol, thus setting dissimilar cleavable sites before assembling the two parallel strands. Infrared analysis unequivocally proved the formation of the isocyanide group, according to the band around 2100 cm^−1^. For the Ugi reaction to be carried out on resin, preformation of the imine is achieved in solution by stirring the Alloc mono‐protected diamine and the aldehyde component in DCM/MeOH 1:1 for 1 h. The mixture is added together with Fmoc‐d‐Pro‐OH (both in four‐fold excess) to the isocyano‐resin and the reaction is shaken for maximum 1 day at room temperature. The success of the Ugi reactions was assessed by RP‐HPLC/ESI‐MS analysis after acidic mini‐cleavage and by quantitative cleavage of the Fmoc group and determination of the absorption at 301 nm (see the Supporting Information). This latter analysis also enabled the calculation of the loading of the different peptide‐functionalized resins **1**–**4**, which is crucial for the subsequent solid‐phase peptide synthesis (SPPS). All four types of amino‐alkyl‐modified resin proved suitable to be converted into isonitriles and to perform the subsequent Ugi reactions, thus providing optimum flexibility with respect to solid support and cleavage sensitivity.

**Scheme 2:**
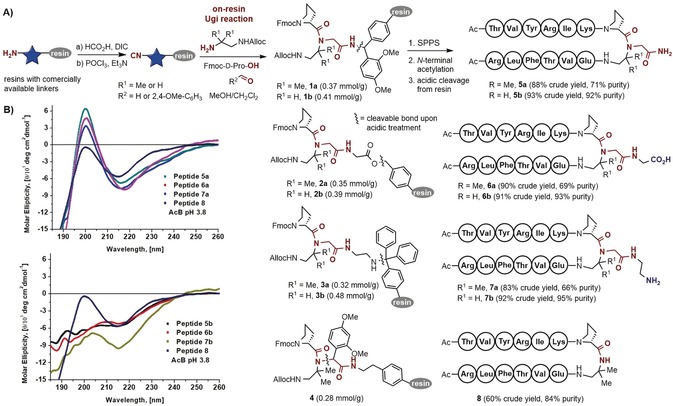
A) Solid‐phase synthesis of parallel β‐sheets via multicomponent assembly and resin anchoring of the N‐alkylated turn‐inducing moiety. B) Circular dichroism spectra of the peptides evaluating their parallel β‐sheet content. AcB: acetate buffer, DIC: diisopropylcarbodiimide, SPPS: solid‐phase peptide synthesis.

Three degrees of orthogonality were employed during the assembly of the two peptide strands using the Fmoc/*t*Bu methodology. Initial cleavage of the Fmoc group enabled the growth of the upper strand, keeping the Alloc‐protected residue unaffected. Removal of this latter protecting group and assembly of the lower strand completed the synthesis of peptides **5**–**8** in good overall yield and purity of the crude products (Scheme 1). The amino acid sequence chosen for peptides **5**–**8** follows some previously established criteria.^7^, ^8^, ^16^ These are, a) having an intrinsic propensity for β‐sheet formation, b) choosing positions to enable favorable (hydrophobic or electrostatic) interactions between paired residues in each strand, and c) adding some basic amino acids to generate positive charges and therefore reduce aggregation.

As shown in Scheme 2, parallel β‐sheets bearing a carboxamide (**5 a**,**b**), a carboxylic acid (**6 a**,**b**), and an amine (**7 a**,**b**) were produced, either with or withou the geminal dimethyl groups in the diamine residue. Thus, we aimed at finding out whether the turn‐inducing capacity of the N‐alkylated peptidomimetic fragment is sufficient to fold the peptides into parallel β‐sheets without the positive influence of the Thorpe–Ingold effect provided by the geminal methyl substituents. The versatility of the MCR method also enabled the facile synthesis of the non‐N‐alkylated β‐sheet, peptide **8**, using the acid‐labile 2,4‐dimethoxyphenyl substituent^17^ (arising from the aldehyde component), which allows the cleavage at that specific position.

Scheme 2 B (upper panel) shows a comparison of the circular dichroism (CD) spectra in acetate buffer (AcB) of the N‐alkylated peptides **5 a**, **6 a**, and **7 a** with the non‐N‐alkylated one (**8**), all based on the DADME residue. The increment in the intensity of the maximum at 198 nm and the minimum at 215 nm, in peptides **5 a**, **6 a**, and **7 a** suggests that the N‐alkylation of the turn‐inducing moiety leads to a higher β‐sheet population. It is plausible to accept a positive effect of the additional amide N‐alkylation on the parallel β‐sheet‐folding propensity; similar to its role in antiparallel β‐sheets (β‐hairpins) when a l‐Pro is added in the turn following the key d‐Pro residue.^2^ It is noteworthy that there are no significant differences between the N‐alkylated β‐sheet peptides **5 a**, **6 a**, and **7 a,** bearing either neutral or charged moieties as part of the turn N‐modification. This indicates that the N‐alkylation, and not the nature of the functionality, plays a positive effect on the folding. On the other hand, comparison of the CD spectra (Scheme 2 B, lower panel) of the N‐alkylated peptides **5 b**, **6 b**, and **7 b** without the additional geminal methyl groups to reference peptide **8** reveals loss of β‐sheet content. Indeed, the conformational preorganization imposed by the geminal methyl groups of the d‐Pro‐DADME turn is crucial for β‐sheet folding in aqueous solution, regardless of the presence of an N‐alkylation.

To our knowledge, there are no methods available for labeling or ligating a β‐sheet peptide without affecting the strand termini or side chains, which are known to be crucial for the β‐sheet stability. In this regard, the capability of the MCR approach to easily functionalize a parallel β‐sheet without modifying the strands shows great prospect in the pursuit of biological applications. To exemplify this possibility, the commercially available cysteamine‐trityl resin was converted into the isocyano‐resin and subjected to the Ugi reaction to assemble the resin‐linked d‐Pro‐DADME fragment **9**. As shown in Scheme 3, subsequent assembly of the two peptide strands followed by mild acidic cleavage from the resin led to the thiol‐functionalized parallel β‐sheet **10** in good yield and moderate purity. Purified peptide **10** was readily conjugated to the maleimide‐functionalized dansyl tag **11** in presence of tris(2‐carboxyethyl)phosphine (TCEP) to furnish the fluorescently labeled parallel β‐sheet **12** in excellent yield. The CD spectra of both peptides **10** and **12** show the characteristic β‐sheet pattern described above, confirming that the type of functionality attached to the turn N‐alkylation does not significantly affect the β‐sheet content in water.

**Scheme 3:**
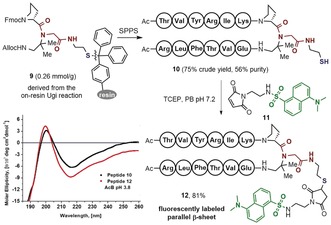
Synthesis of a thiol‐functionalized parallel β‐sheet peptide, its conjugation to a fluorescent label, and circular dichroism analysis. PB: phosphate buffer; TCEP: tris(2‐carboxy‐ethyl)phosphine.

Further to the CD evidence for a higher β‐sheet content in Ugi‐peptides, we sought confirmation by NMR spectroscopy. Initially, the NMR analysis was employed to assess the tendency of the peptides to aggregate and whether the N‐alkylation functionality has an influence on it. This was accomplished following a methodology used by Gellman to evaluate this phenomenon in model parallel β‐sheets.^7^, ^8^ Thus, the NMR spectra of all peptides bearing the d‐Pro‐DADME turn fragment were acquired at 2.5 mm concentration in 9:1 H_2_O/D_2_O containing 100 mm of sodium acetate buffer (pH 3.8). Peptide **7 a**, which bears an additional positive charge, shows the sharpest and most defined signal lines in the ^1^H NMR spectrum, with no change in the chemical shift and remaining stable in solution (i.e., without precipitation) even after aging for 6 months. The superposition of three mono‐dimensional ^1^H NMR spectra of peptide **7 a** at concentrations 0.5 mm, 1 mm, and 2.5 mm, showed no changes in the in the chemical shifts of the amide hydrogens, indicating absence of self‐association (see the Supporting Information). In contrast, the ^1^H NMR spectrum of peptide **6 a**, with a carboxylic acid at the N‐alkylation moiety, shows broader signal lines and signs of precipitation after aging. Interestingly, peptides **5 a** and **8** exhibit signal lines broader than **7 a**, but no sign of aggregation after aging in solution for 6 months.

Chemical shift deviation (CSD) of the amino acid α‐protons of a well‐structured peptide with respect to the random coil state, i.e., Δ*δ*_CαH_=*δ*_CαH_ (observed)−*δ*_CαH_ (random coil) is a powerful tool in secondary structure determination. It is known that having a set of three or more residues with *δ*_αH_ shifted downfield by ≥0.1 ppm can be taken as evidence of β‐sheet conformation.^18^ Accordingly, we focused on assessing the influence of the N‐alkylation in the β‐sheet content by comparing the CSD of peptides **7 a** and **8**, using the random coil data from the original report by Richards’ group.^18^ As shown in Figure 1 A, the Δ*δ*_CαH_ data confirm that both peptides occur as parallel β‐sheets in aqueous buffer, with most of the residues having a Δ*δ*_CαH_ ≥0.1 ppm. Importantly, the higher Δ*δ*c_αH_ values for the N‐alkylated peptide **7 a** corroborate the evidence provided by the CD spectra, that this peptide has a better structured β‐sheet conformation than the non‐N‐alkylated one.

**Figure 1:**
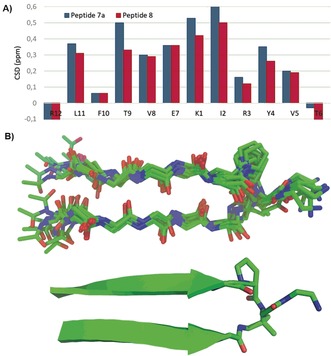
A) Comparison of the chemical shift deviation (CSD) of peptides **7 a** and **8** in aqueous acetate buffer. B) NMR‐structure of peptide **7 a** based on NOE and dihedral angle constraints.

To determine the NMR structure of peptide **7 a** in aqueous solution, the full resonance assignment was accomplished to look for the presence of NOEs between nonadjacent residues. Such interstrand NOEs are decisive to corroborate the β‐sheet conformation and to construct the NOE‐restrained dynamics of this peptide. Figure 1 B shows the NMR structure of **7 a**, built using 11 NOEs between adjacent residues, six interstrand NOEs and 19 dihedral angle restrictions. Only one rotamer of the N‐alkylated (tertiary) amide is observed. The superposition of the ten lowest‐energy conformations displays the good alignment of the two parallel strands, confirming the well‐structured nature of this β‐sheet (RMSD among backbone atoms 0.64±0.02 Å). As seen in other non‐cyclic parallel β‐sheets,^7^ the major deviations were observed at the N‐termini, but the interstrand NOE between the penultimate Val and Leu residues confirms the well‐developed β‐sheet conformation alongside the two strands. Besides the steric bias provided by the turn N‐alkylation that helps to bend the peptide backbone, this modification removes the possibility of γ‐turn formation, which would not favor folding into a parallel β‐sheet due to its tight nature.

To evaluate the stability of the N‐alkylated β‐sheet structures, we decided to compare the CD spectra of peptides **7 a** and **8** in aqueous solution at different temperatures. Figure 2 depicts the CD spectra at 15, 35, 55, and 75 °C, clearly showing that the N‐alkylated peptide **7 a** maintains the parallel β‐sheet conformation even at higher temperatures, while non‐N‐alkylated **8** shows a steep decrease of the β‐sheet population already at 55 °C. This corroborates the greater capacity of the N‐alkylated turn to keep the desired fold intact, not only at physiological temperature but also under thermic stress.

**Figure 2:**
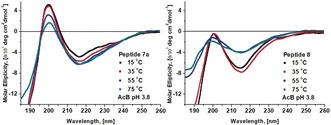
Comparison by circular dichroism of the β‐sheet stability of peptides **7 a** and **8** in aqueous acetate buffer at different temperatures.

In conclusion, we have developed an efficient multicomponent approach for the on‐resin assembly of N‐alkylated parallel β‐sheet peptides with improved stability and varied functionalization. The key step in this methodology is the Ugi four‐component reaction that allows the construction of the N‐alkylated turn fragment in one step by simultaneously incorporating d‐Pro and DADME residues onto the isocyano‐modified resins. The procedure shows great versatility, as it enables not only the variation of the functionalization of the N‐alkylation moiety at will, but also the access to non*‐*N‐alkylated β‐sheets in a more convergent manner than previously described. CD analysis confirmed the positive effect of the turn N‐alkylation on β‐sheet stability, even at high temperatures. Furthermore, the NMR structure of a selected N‐alkylated peptide confirmed the formation of a well‐structured parallel β‐sheet in water. Overall, we demonstrated that the functionality attached to the turn N‐alkylation can be tuned to reduce aggregation (adding positive charges) or used for fluorescent labeling without modification of the strand backbone and side chains and without affecting the β‐sheet stability. By providing an efficient, all‐on resin approach toward stable β‐sheet‐bearing tunable functionalizations, we open a variety of possibilities for the—eventually bioorthogonal—construction of mini‐proteins based either on multiple parallel β‐sheets or on a combination of parallel β‐sheets with other types of secondary structures, such as α‐helices and antiparallel β‐hairpins.

## Conflict of interest

The authors declare no conflict of interest.

## Supporting information

As a service to our authors and readers, this journal provides supporting information supplied by the authors. Such materials are peer reviewed and may be re‐organized for online delivery, but are not copy‐edited or typeset. Technical support issues arising from supporting information (other than missing files) should be addressed to the authors.

Supplementary
