# Supplementary material for: Improved Stability and Tunable Functionalization of Parallel β‐Sheets via Multicomponent N‐Alkylation of the Turn Moiety
Source: Angew Chem Int Ed Engl. 2019 Dec 4;59(1):259–63. doi: 10.1002/anie.201912095 (PMC6973259; doi:10.1002/anie.201912095)
Supplement: Supplementary file 1 — Supplementary [file ANIE-59-259-s001.pdf]

Angewandte Chemie
Eine Zeitschrift der Gesellschaft Deutscher Chemiker

Supporting Information

Improved Stability and Tunable Functionalization of Parallel β-Sheets via Multicomponent N-Alkylation of the Turn Moiety

Manuel G. Ricardo, Celia G. Moya, Carlos S. Pérez, Andrea Porzel, Ludger A. Wessjohann, and Daniel G. Rivera**

anie_201912095_sm_miscellaneous_information.pdf

Supporting Information (SI)

Abbreviations 2
General Information 2
Synthesis of isocyano-resins 3
Solid-phase synthesis of the resin-linked turn inducers 7
On-resin synthesis of parallel beta-sheet peptides 11
General methods for Solid Phase Peptide Synthesis (SPPS) 11
Description of the general synthetic procedure 11
Circular dichroism analysis 30
NMR analysis and simulated annealing 33

Abbreviations

AcB, acetate buffer; Alloc, allyloxycarbonyl; 2CT, 2-chlorotrityl; DADME, 1,2-diamino-1,1-dimethylethane; DIC, diisopropylcarbodiimide; DIPEA, diisopropylethylamine; DMF, dimethylformamide; ESI-MS, electrospray ionization mass spectrometry; DSS, 2,2-dimethyl-2-silapentane-5-sulfonate; FA, formic acid; Fmoc, fluorenylmethyloxycarbonyl; HOBt, hydroxybenzotriazol, HR-MS, high resolution mass spectrometry; IR, infrared; MeOH, methanol; PB, phosphate buffer; RP-HPLC, reversed-phase high-performance liquid chromatography; RT, room temperature; SPPS, solid phase peptide synthesis; *t*Bu, *tert*-butyl; TCEP, 2-(carboxyethyl)phosphine hydrochloride; TFA, trifluoroacetic acid; TFE, 2,2,2-trifluoroethanol; TG-S-RAM, Tentagel resin Rink amide; THF, tetrahydrofuran; TIS, triisopropylsilane; TLC, thin layer chromatography; TMS, tetramethyl silane; Trt, triphenylmethyl.

General Information

All starting materials were purchased from commercial sources and used without further purification. ^{1}H NMR and ^{13}C NMR spectra were recorded on an Agilent (Varian) VNMRS 600 NMR spectrometer at 599.83 MHz and 150.83 MHz, respectively. Chemical shifts (δ) are reported in ppm relative to the 2,2-dimethyl-2-silapentane-5-sulfonate, DSS (^{1}H NMR) and to the solvent signal (^{13}C NMR). IR spectra were obtained on a Thermo Nicolet 5700 FT-IR spectrometer. High resolution ESI-MS data was obtained from a Bruker Apex III Fourier transform ion cyclotron resonance (FT-ICR) mass spectrometer equipped with an InfinityTM cell, an RF-only hexapole ion guide and an external electrospray ion source (Agilent, off axis spray). Analytical RP-HPLC analysis was performed with an Agilent 1100 system in a reverse-phase C18 column (4.6 × 150 mm, 5 µm) with a PDA detector. A linear gradient from 5% to 60% of solvent B in solvent A over 20 min at a flow rate of 0.8 mLmin^{-1} was used. The preparative purification was performed on Knauer 1001 system with UV detector K-2501. Separation was achieved using an RP C18 column (25 × 250 mm, 25 µm). A linear gradient from 15% to 40% of solvent B in solvent A over 20 min at a flow rate of 5 mLmin^{-1} was used. Detection was accomplished at 210 nm. Solvent A: 0.1% (*v*/*v*) formic acid (FA) in water. Solvent B: 0.1% (*v*/*v*) FA in acetonitrile. Circular dichroism spectra were collected on a Jasco J-815 spectropolarimeter equipped with a temperature controller at 25 ºC over the wavelength range 260-185 nm, using standard measurement parameters: 50 nm/sec speed, 16 accumulations, 1 mm path length. Flash column chromatography was carried out using Merck silica gel 60 (0.015-0.040 nm) and analytical thin layer chromatography (TLC) was performed using Merck silica gel 60 F254 aluminum sheets.

Synthesis of isocyano-resins

General on-resin formylation-dehydration procedure: The free amino group-bound resin (0.72 mmol) is placed in a 10 mL solid-phase reaction vessel and pre-swelled with dry DCM (2×8 mL). Formic acid (0.13 mL, 3.6 mmol, 5 equiv) is pre-activated with DIC (0.54 mL, 3.6 mmol, 5 equiv) during 4 min in DMF (5 mL) in a 4 mL vessel. This mixture is transferred into the resin, stirred for 2 h and checked by Kaiser test (if is not complete, the procedure is repeated once). The resin is washed with DMF (3×8 mL) and DCM (2×8 mL). The vessel is connected with a gentle stream of N_2(g), dry DCM (8 mL) is added to the mixture and Et_3N (1.5 mL, 10.8 mmol, 15 equiv). $POCl_3$ (0.33 mL, 3.5 mmol, 5 equiv) is added dropwise to the reaction mixture and with the stream of nitrogen the system is stirred for 4 hours. The resin is sequentially washed with DCM (3×8 mL) and DMF (3×8 mL), dried in a desiccator for 1 day and stored at -20 ºC. The isocyanide formation is checked by infrared spectroscopy.

Isocyanide-functionalized MBHA resin

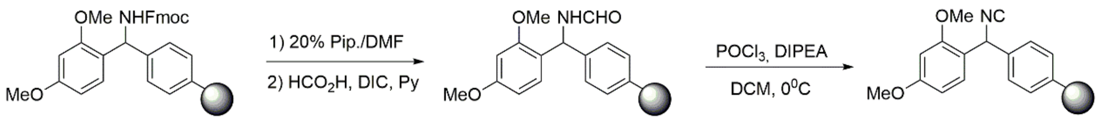

The MBHA resin (1.0 g, 0.72 mmol/g) was subjected to Fmoc removal, then formylated and dehydrated according to the general procedure described above.

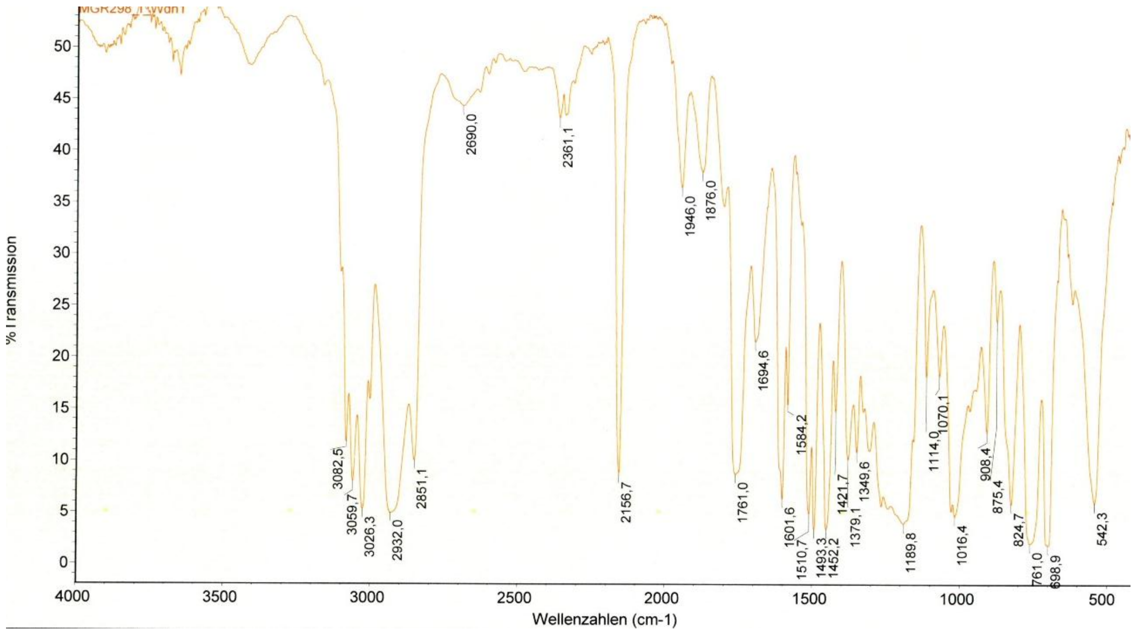

Figure S1. Infrared spectrum of the isocyanide-functionalized MBHA resin.

Isocyanide-functionalized Gly-Wang Resin

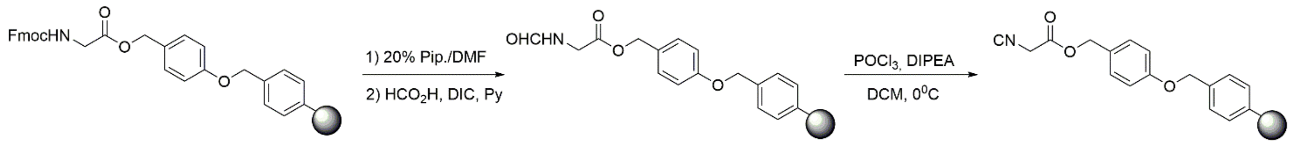

Fmoc-Gly-Wang resin (1.2 g, 0.6 mmol/g) was pre-swelled with DCM (2×8 mL) and subjected to Fmoc removal, then formylated and dehydrated according to the general procedure described above.

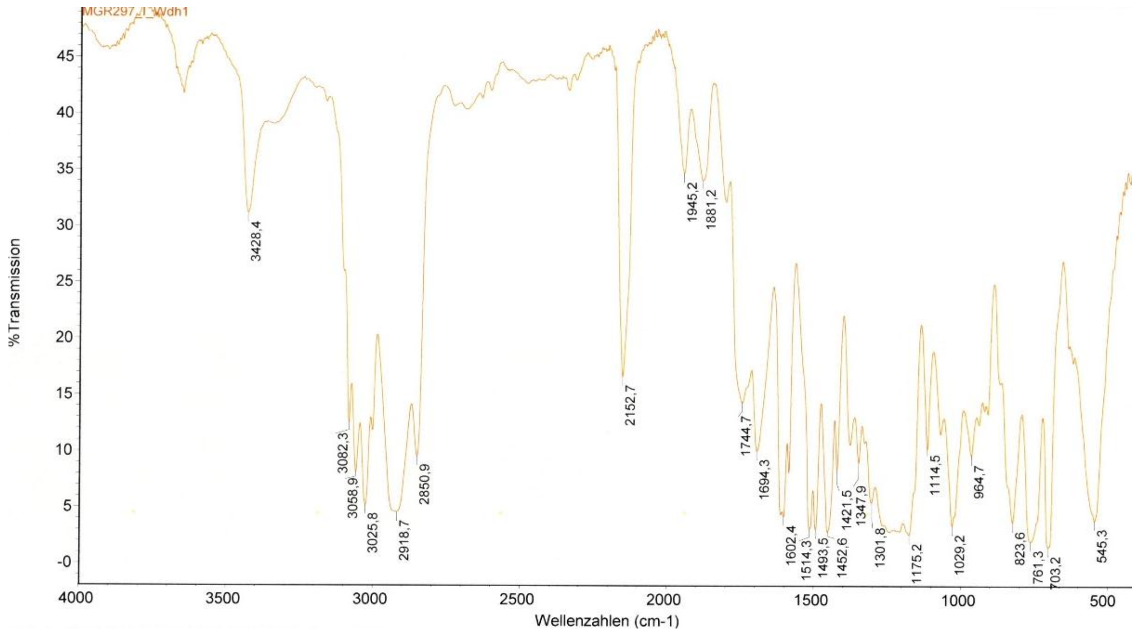

Figure S2. Infrared spectrum of the isocyanide functionalized Gly-Wang resin.

Isocyanide-functionalized ethylendiamine-trityl resin

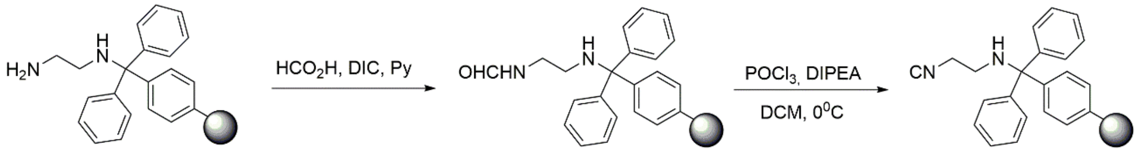

The 1,2-ethanodiamine-trityl resin (1.2 g, 0.6 mmol/g) was subjected to formylation and dehydration according to the general procedure described above.

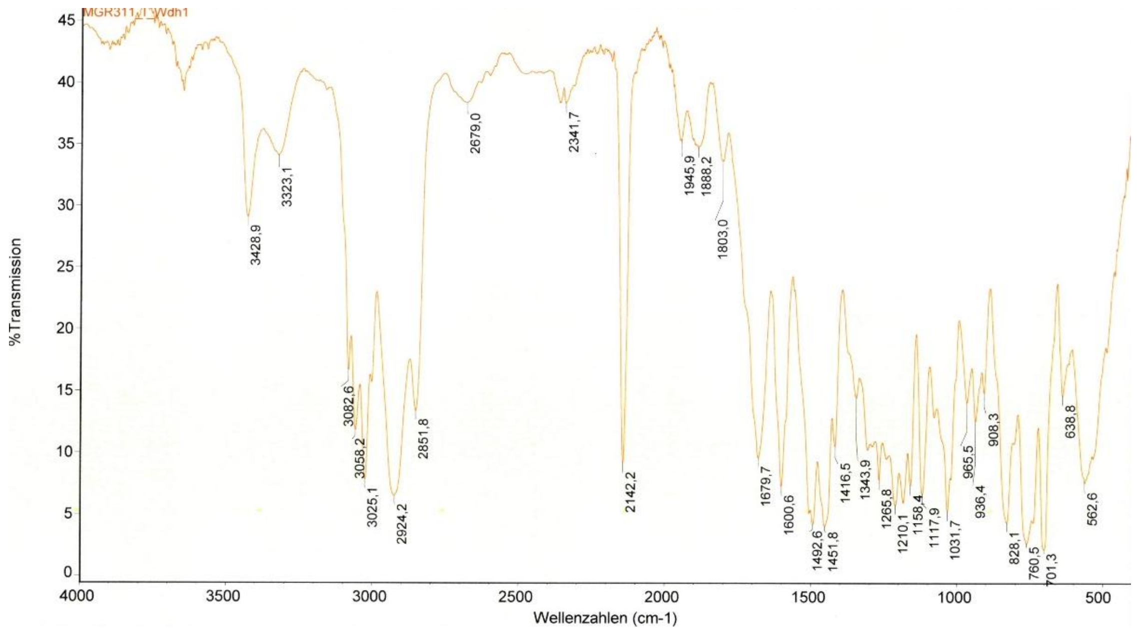

Figure S3. Infrared spectrum of the isocyanide-functionalized ethylenediamine-Trt resin.

Isocyanide-functionalized 2-aminoetyl polystyrene resin

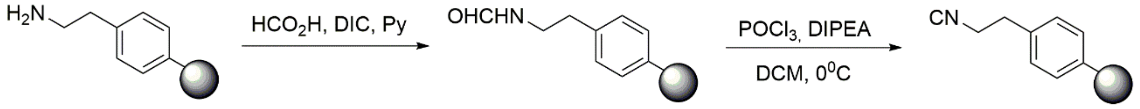

The 2-aminoethyl polystyrene resin (0.72 g, 1 mmol/g) was subjected to formylation and dehydration according to the general procedure described above.

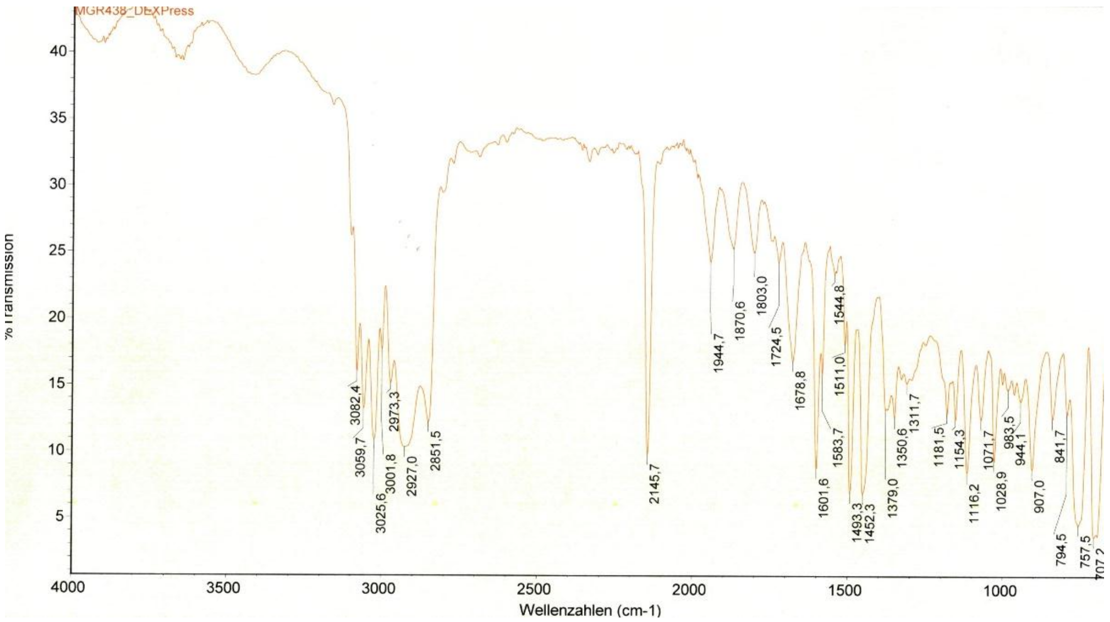

Figure S4. Infrared spectrum of the isocyanide-functionalized 2-aminoethyl polystyrene resin.

Isocyanide-functionalized cysteamine-trityl resin

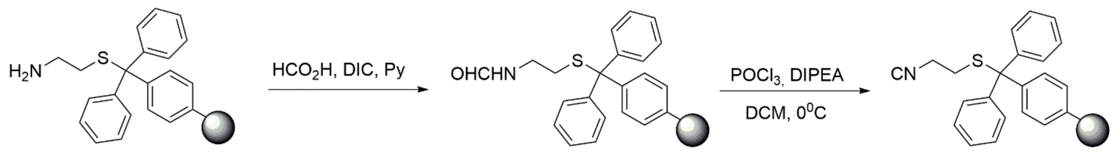

The 1,2-ethanodiamine-trityl resin (1.2 g, 0.6 mmol/g) was subjected to formylation and dehydration according to the general procedure described above.

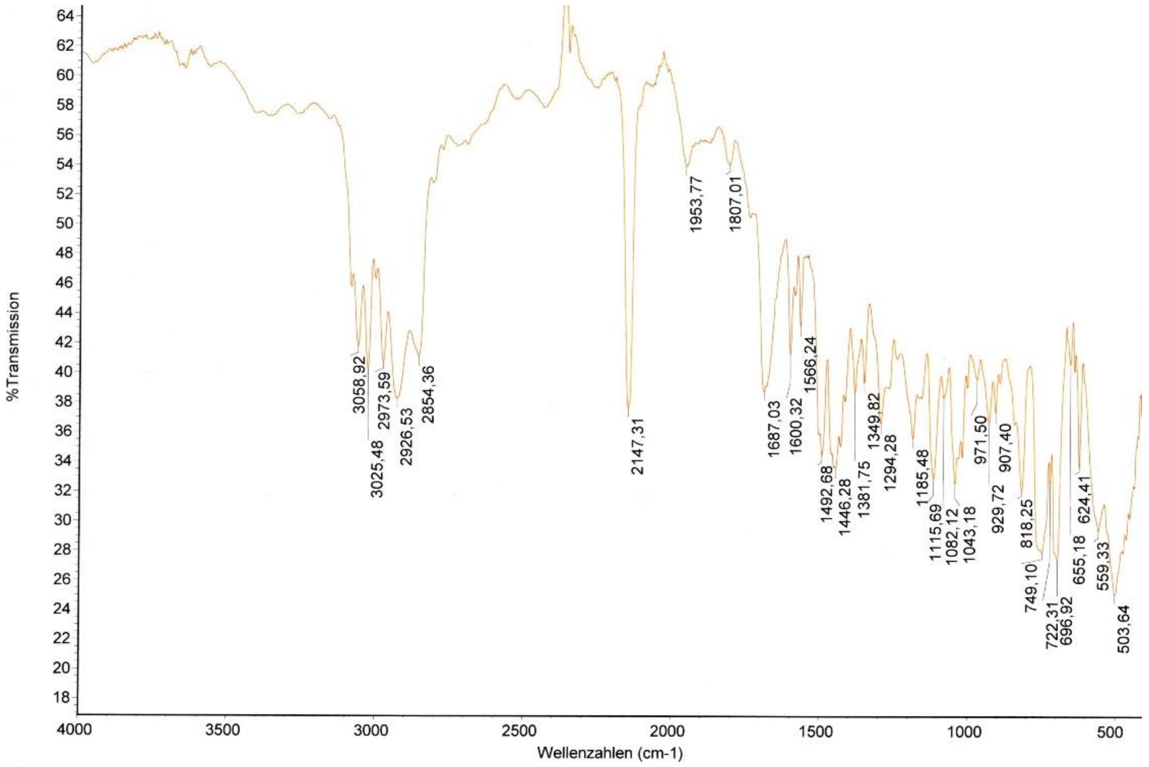

Figure S5. Infrared spectrum of the isocyanide-functionalized cysteamine-Trt resin.

Solid-phase synthesis of the resin-linked turn inducers

On-resin Ugi reaction: In a 4 mL vial, 2-alloc-DADME (4 equiv) or 1-alloc-ethylendiamine (both diamines monoprotected according to literature procdures[1,2]) are mixed with a suspension of paraformaldehyde (4 equiv) in MeOH (1 mL) and stirred for 1 h. Fmoc-D-Pro-OH (4 equiv) is added to the reaction mixture, 1 mL of DCM is added and the resulting mixture is transferred into a solid-phase vessel containing the isocyano-resin. The reaction mixture is left shaking at room temperature for 24 h. The excess of reagents is removed by washing the beads sequentially with DMF (3×1 min), DCM (3×1 min) and Et_2O (2×1 min). The formation of the *N*-alkylated D-Pro-DADME turn is checked by ESI-MS and RP-HPLC monitoring after mini-cleavage. Finally, the resin loading is determined spectrophotometrically by UV absorption of the Fmoc group at 301 nm as described in the following.

Loading measurement: The loaded resin (5-10 mg) in an Eppendorf tube is mixed with 20% piperidine/DMF (1 mL) and stirred for 20 min. The resulting suspension is centrifuged and 100 µL are transferred to a vessel containing DMF (10 mL). Finally, part of the above solution is transferred into a 1 mL cuvette and the absorbance is measured at 301 nm. The same procedure is repeated for the reference (same composition solution but without the resin). The loading of the resin is calculated by $L(\text{mg mmol}^{-1}) = [101\times(\text{Absorbance})]/[7.8\times(\text{weight in mg})]$.

On-resin synthesis of the *N*-alkylated peptide fragment 1a

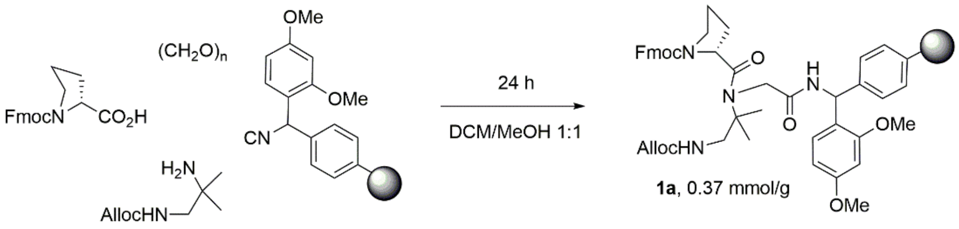

The isocyanide-functionalized ethylendiamine-Trt resin (0.3 mmol), Fmoc-D-Pro-OH (0.4 g, 1.2 mmol), paraformaldehyde (36 mg, 1.2 mmol) and 2-Alloc-DADME (0.2 g, 1.2 mmol) were reacted according to the Ugi reaction general procedure described above to afford the peptide fragment **1a**. Final loading: 0.37 mmol/g.

[1] M. Hurevich, *et al.*, *J. Pept. Sci.* **2010**, *16*, 178.
[2] J. D. Fisk, D. R. Powell, S. H. Gellman, *J. Am. Chem. Soc.* **2000,** *122*, 5443.

On-resin synthesis of the *N*-alkylated peptide fragment 1b

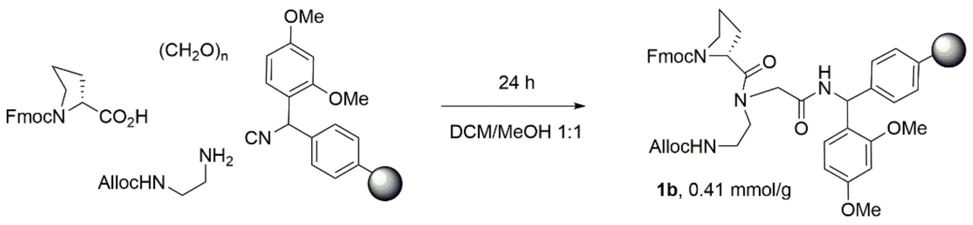

Isocyanide-functionalized ethylendiamine-Trt resin (0.3 mmol), Fmoc-D-Pro-OH (0.4 g, 1.2 mmol), paraformaldehyde (36 mg, 1.2 mmol) and 1-Alloc-ethylendiamine (0.17 g, 1.2 mmol) were reacted according to the Ugi reaction general procedure described above to afford the peptide fragment **1b**. Final loading: 0.41 mmol/g.

On-resin synthesis of the *N*-alkylated peptide fragment 2a

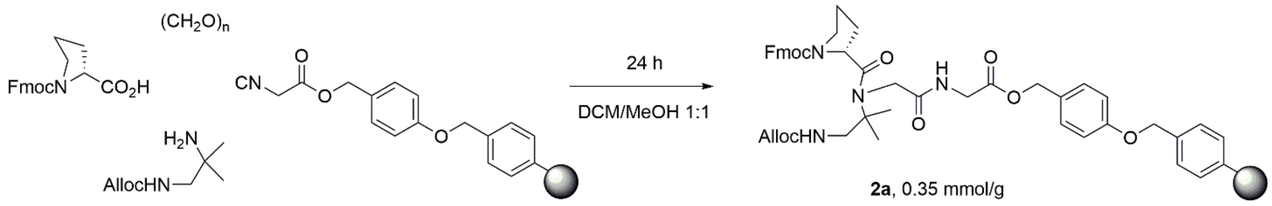

Isocyanide-functionalized Gly-Wang resin (0.3 mmol), Fmoc-D-Pro-OH (0.4 g, 1.2 mmol), paraformaldehyde (36 mg, 1.2 mmol) and 2-Alloc-DADME (0.2 g, 1.2 mmol) were reacted according to the Ugi reaction general procedure described above to afford the peptide fragment **2a**. Final loading: 0.35 mmol/g.

On-resin synthesis of the *N*-alkylated peptide fragment 2b

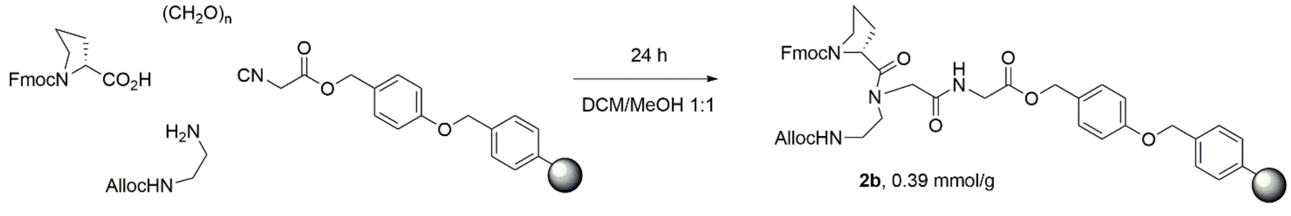

Isocyanide-functionalized Gly-Wang resin (0.3 mmol), Fmoc-D-Pro-OH (0.4 g, 1.2 mmol), paraformaldehyde (36 mg, 1.2 mmol) and 1-Alloc-ethylendiamine (0.17 g, 1.2 mmol) were reacted according to the Ugi reaction general procedure described above to afford the peptide fragment **2b**. Final loading: 0.39 mmol/g.

On-resin synthesis of the *N*-alkylated peptide fragment 3a

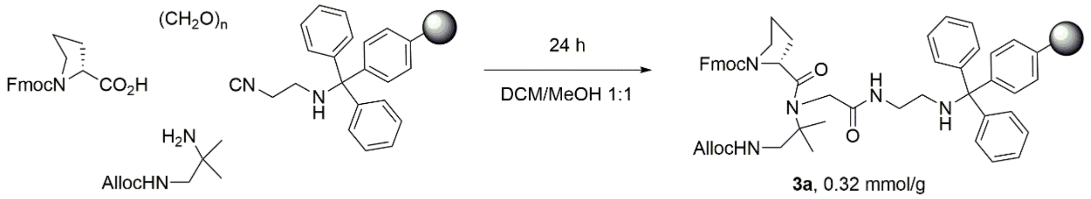

Isocyanide-functionalized ethylendiamine-Trt resin (0.3 mmol), Fmoc-D-Pro-OH (0.4 g, 1.2 mmol), paraformaldehyde (36 mg, 1.2 mmol) and 2-Alloc-DADME (0.2 g, 1.2 mmol) were reacted according to the Ugi reaction general procedure described above to afford the peptide fragment **3a**. Final loading: 0.32 mmol/g.

On-resin synthesis of the *N*-alkylated peptide fragment 3b

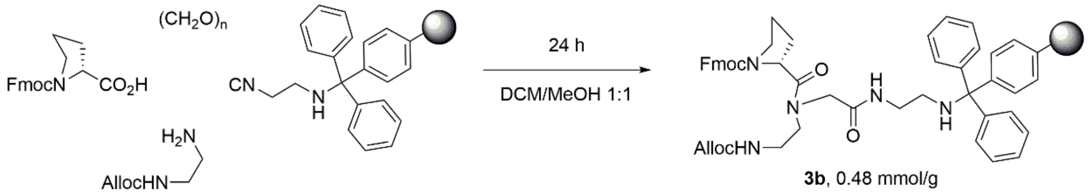

Isocyanide-functionalized ethylendiamine-Trt resin (0.3 mmol), Fmoc-D-Pro-OH (0.4 g, 1.2 mmol), paraformaldehyde (36 mg, 1.2 mmol) and 1-Alloc-ethylendiamine (0.17 g, 1.2 mmol) were reacted according to the Ugi reaction general procedure described above to afford the peptide fragment **3b**. Final loading: 0.48 mmol/g.

On-resin synthesis of the peptide 4

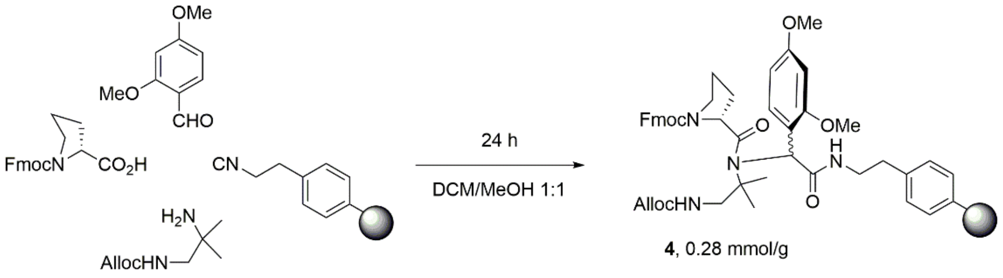

Isocyanide-functionalized 2-aminoethyl polystyrene resin (0.3 mmol), Fmoc-D-Pro-OH (0.4 g, 1.2 mmol), 2,4-dimethoxybenzaldehyde (0.2 g, 1.2 mmol) and 2-Alloc-DADME (0.2 g, 1.2 mmol) were reacted according to the Ugi reaction general procedure described above to afford the peptide fragment **4**. Final loading: 0.28 mmol/g.

On-resin synthesis of the *N*-alkylated peptide fragment 9

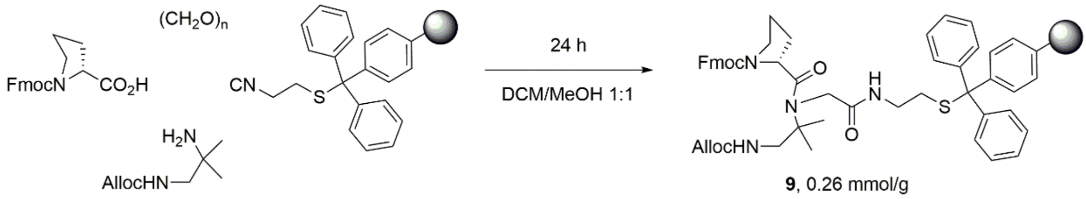

9, 0.26 mmol/g

Isocyanide-functionalized cysteamine-Trt resin (0.3 mmol), Fmoc-D-Pro-OH (0.4 g, 1.2 mmol), paraformaldehyde (36 mg, 1.2 mmol) and 2-Alloc-DADME (0.2 g, 1.2 mmol) were reacted according to the Ugi reaction general procedure described above to afford the peptide fragment **9**. Final loading: 0.26 mmol/g.

On-resin synthesis of parallel beta-sheet peptides

General methods for Solid Phase Peptide Synthesis (SPPS)

Coupling reactions were carried out manually on the resin-linked peptide fragments (0.05 mmol) by a stepwise Fmoc/*t*Bu strategy. *Swelling*: The resin is swelled for 20 min in dichloromethane. *Fmoc removal*: The resin is treated with a solution of 20% piperidine in *N*,*N*-dimethylformamide (DMF) (2×10 min), then washed with DCM (3×1 min) and DMF (2×1 min). *DIC/HOBt coupling*: Fmoc protected amino acid (4.0 equiv) and 1-hydroxybenzotriazole (HOBt) (4.0 equiv) are dissolved in DMF, then diisopropylcarbodiimide (DIC) (4 equiv) is added. The mixture is pre-activated for 5 min, added to the resin, and the system stirred at room temperature until completion indicated by the Kaiser test.[3] Then the resin is washed with DCM (3×1 min) and DMF (2×1 min). *Alloc Removal*: The resin is washed with dry dichloromethane (2×2 min) under a stream of nitrogen. A solution of phenylsilane (20 equiv) in dry dichloromethane and tetrakis(triphenylphosphine)palladium(0) (0.2 equiv) are added to the resin under a continuous stream of nitrogen. The mixture is stirred in the dark for 15 min, and the procedure is repeated once and then washed with 0.5% of sodium diethyldithiocarbamate trihydrate in DMF (5×2 min) and DCM (2×2 min). *Acetylation*: A mixture of acetic anhydride (10 equiv) and DIPEA (10 equiv) in DMF is added to the resin and the mixture is stirred for 30 min at room temperature, then washed with DCM (3×1 min) and DMF (2×1 min). *Cleavage:* The resin is treated with the cocktail TFA/TIS/H2O (95:2.5:2.5). The peptide is precipitated from cold diethyl ether (-20 ºC), then taken up in 1:2 acetonitrile/water and lyophilized.

Description of the general synthetic procedure

Starting from the resin-linked peptide fragment, Fmoc-Lys(Boc)-OH, Fmoc-Ile-OH, Fmoc-Arg(Pbf)-OH, Fmoc-Tyr(OtBu)-OH, Fmoc-Val-OH, Fmoc-Thr(*t*Bu)-OH are sequentially coupled and the *N*-terminus is acetylated according to the procedure described above. Then, the Alloc group is selectively removed and Fmoc-Glu(*t*Bu)-OH, Fmoc-Val-OH, Fmoc-Thr(*t*Bu)-OH, Fmoc-Phe-OH, Fmoc-Leu-OH, Fmoc-Arg(Pbf)-OH are incorporated and the amino group of this strand is also acetylated. The peptide is cleaved, precipitated from the eluate by treatment with cold diethyl ether (-20 ºC), taken up in 1:2 acetonitrile/water and lyophilized.

[3] E. Kaiser, R. L. Colescott, C. D. Bossinger, P. I. Cook, *Anal Biochem*. **1970**, *34*, 595.

Synthesis of peptide 5a

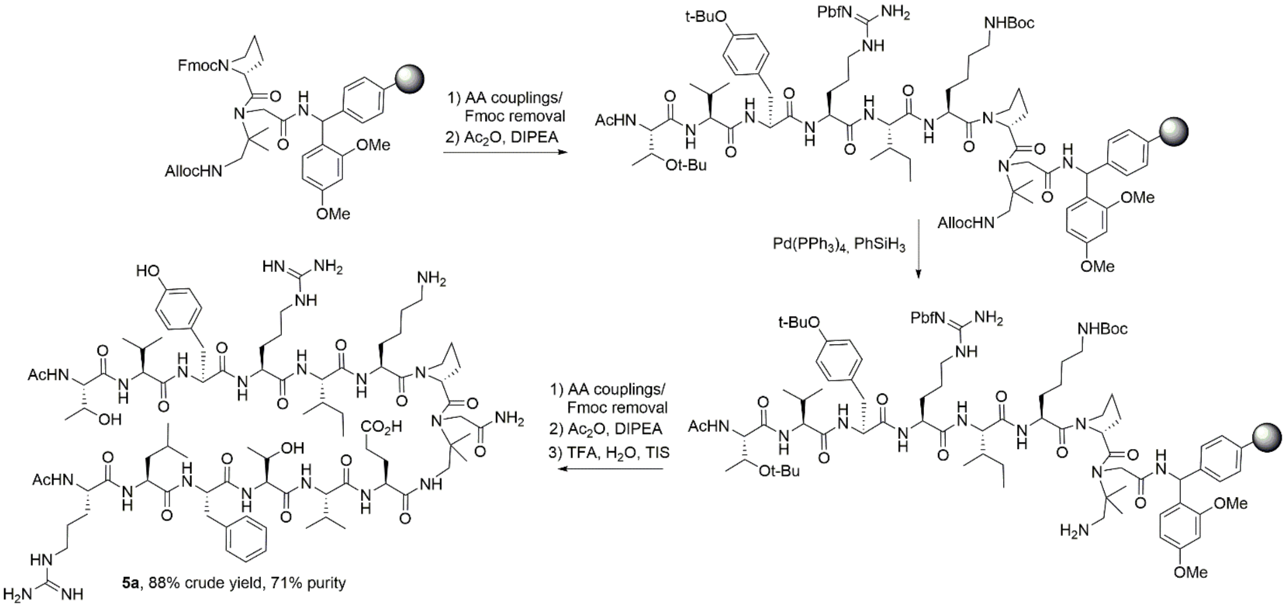

Peptide **5a** was obtained as an amorphous white solid (81 mg, 88% crude yield, 71% purity) starting from **1a** (180 mg, 0.05 mmol) according to the general procedure described above. An analytical sample (20 mg) was purified by preparative RP-HPLC for NMR, CD and HR-MS characterization. R_t = 10.8 min. HR-MS *m/z*: 611.6944 $[M+3H]^{3+}$, calcd for $C_{86}H_{144}N_{23}O_{21}$: 611.6969.

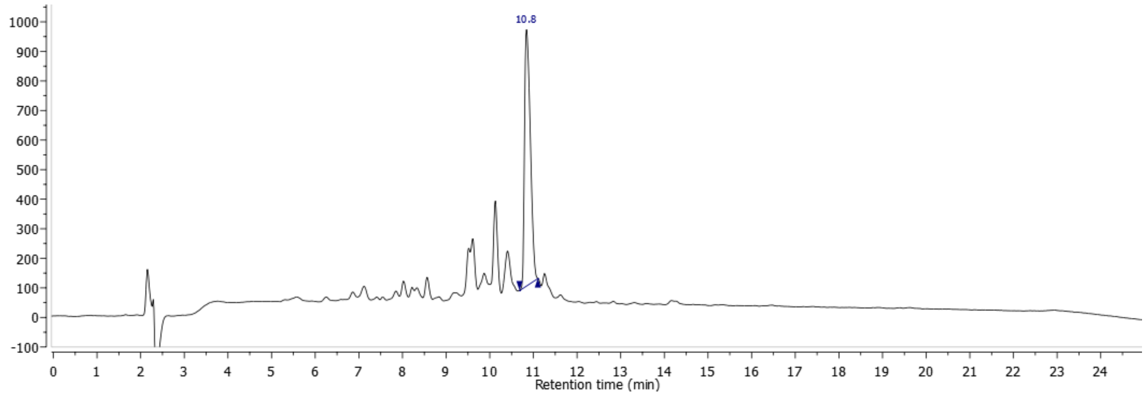

Figure S6. RP-HPLC trace of crude peptide **5a**.

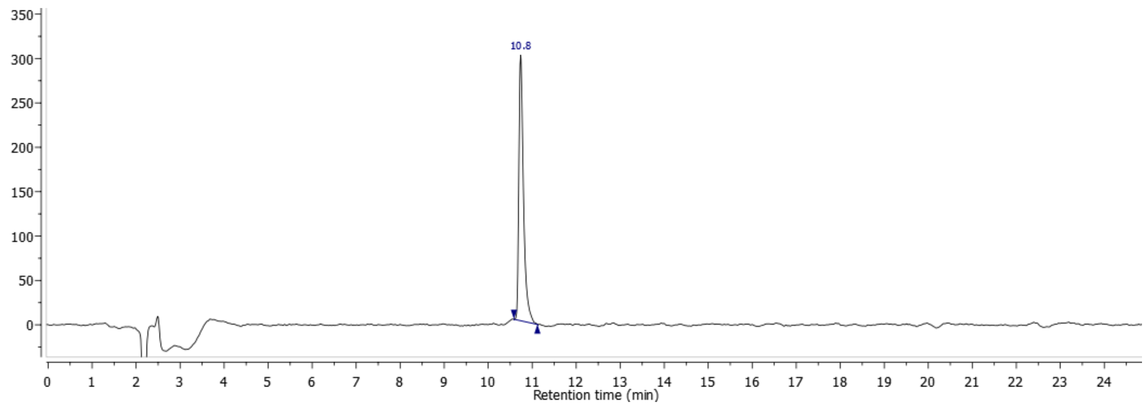

Figure S7. RP-HPLC trace of purified peptide **5a**.

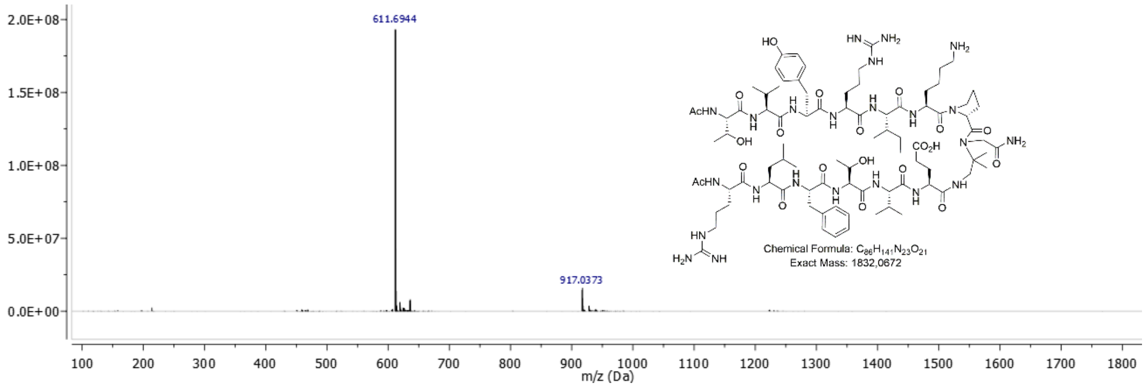

Figure S8. ESI-HRMS of peptide **5a**.

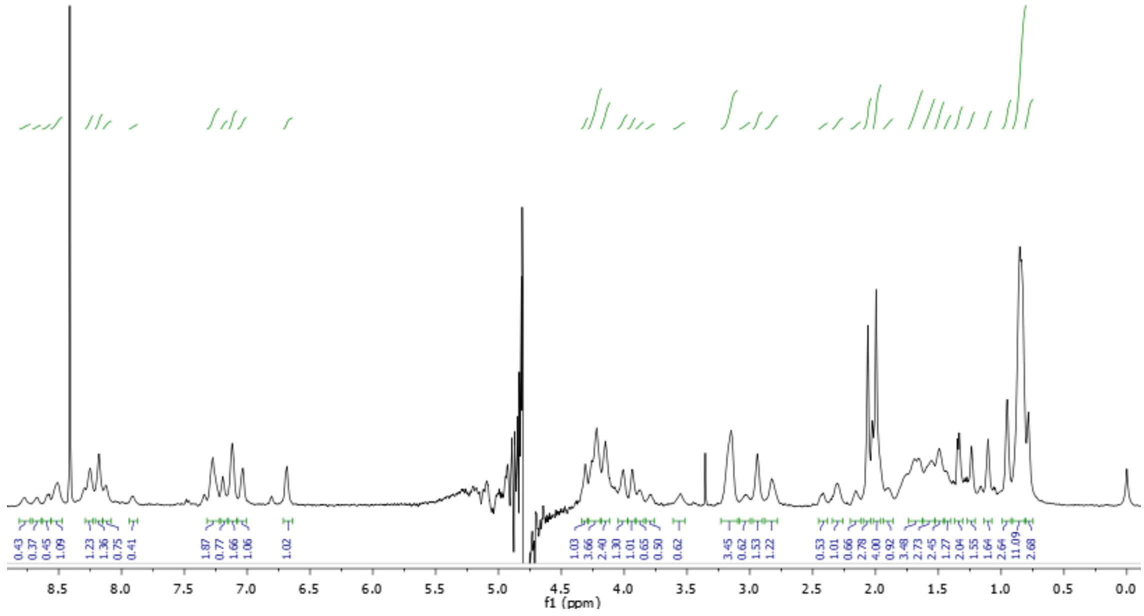

Figure S9. ^{1}H NMR spectrum (600 MHz, 9:1 H_2O:D_2O, 100 mM sodium deuteroacetate buffer, pH 3.8) of peptide **5a**.

Synthesis of peptide 5b

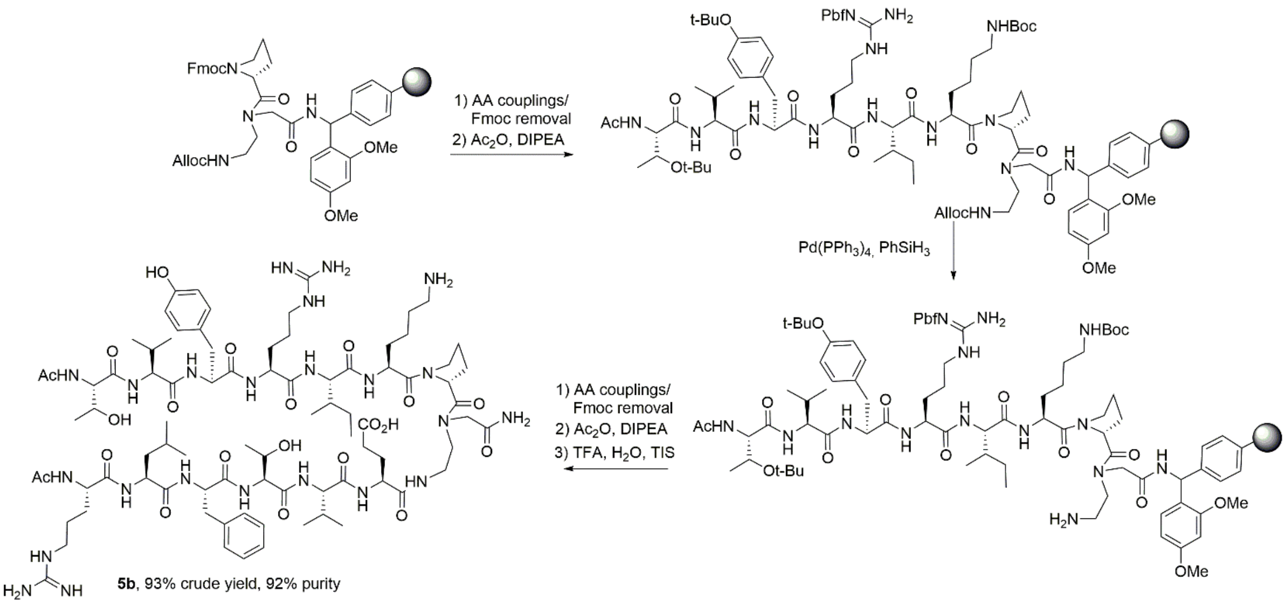

Peptide **5b** was obtained as an amorphous white solid (84 mg, 93% crude yield, 92% purity) starting from **1b** (122 mg, 0.05 mmol)according to the general procedure described above. An analytical sample (20 mg) was purified by preparative RP-HPLC for NMR, CD and HR-MS characterization. R_t = 11.4 min. HR-MS *m/z*: 602.3509 $[M+3H]^{3+}$, calcd. for $C_{84}H_{140}N_{23}O_{21}$: 602.3531.

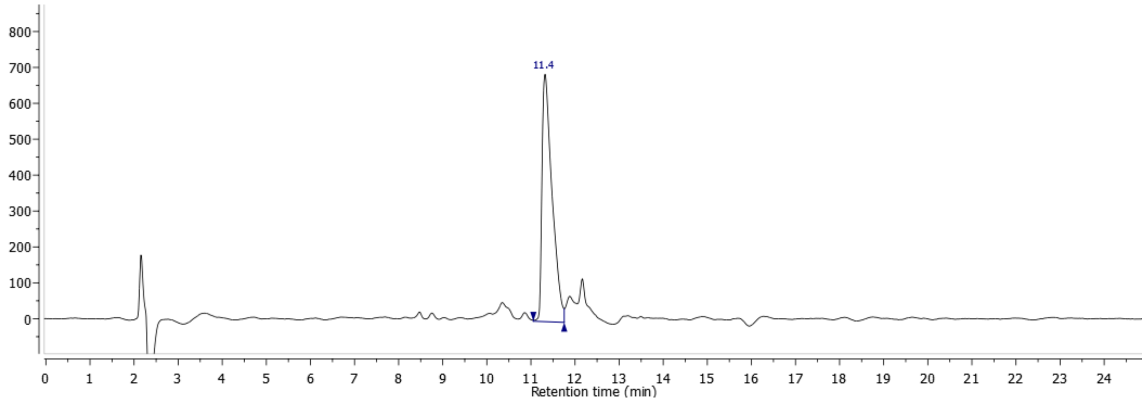

Figure S10. RP-HPLC trace of crude peptide **5b**.

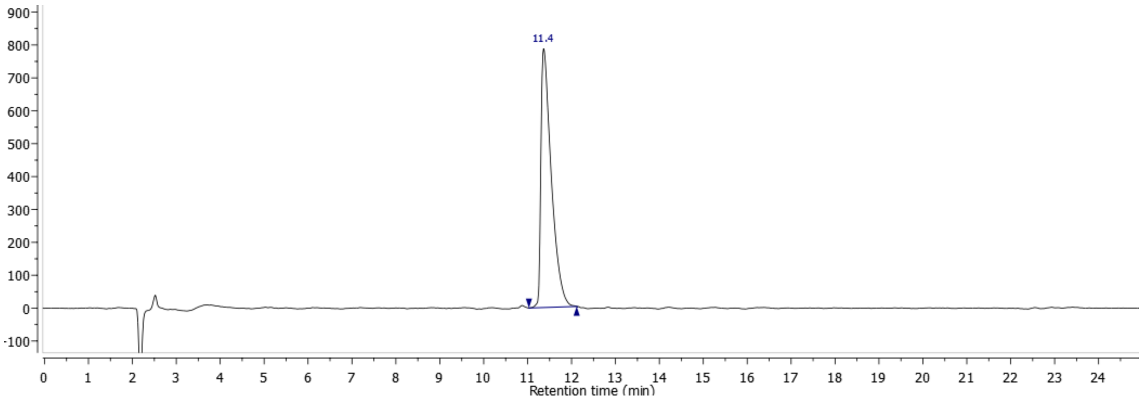

Figure S11. RP-HPLC trace of purified peptide **5b**.

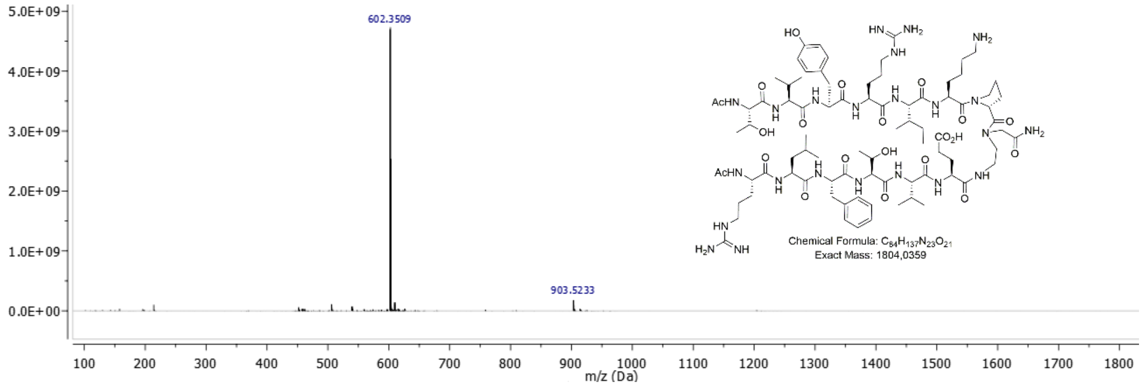

Figure S12. ESI-HRMS of peptide **5b**.

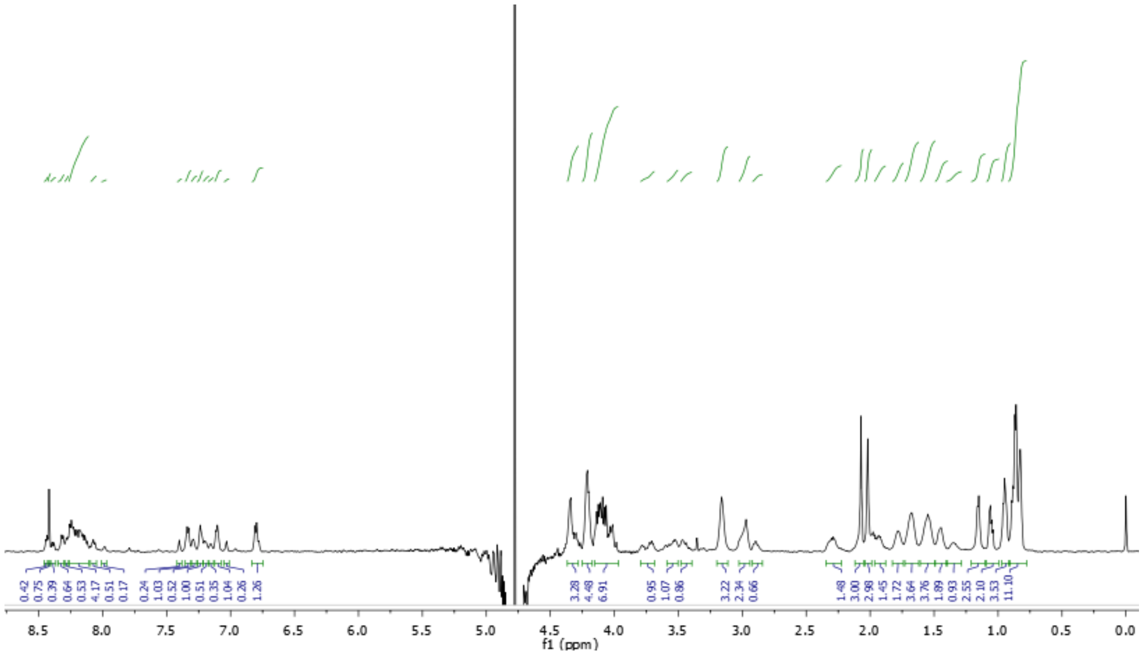

Figure S13. ^{1}H NMR spectrum (600 MHz, 9:1 H_2O:D_2O, 100 mM sodium deuteroacetate buffer, pH 3.80) of peptide **5b**.

Synthesis of peptide 6a

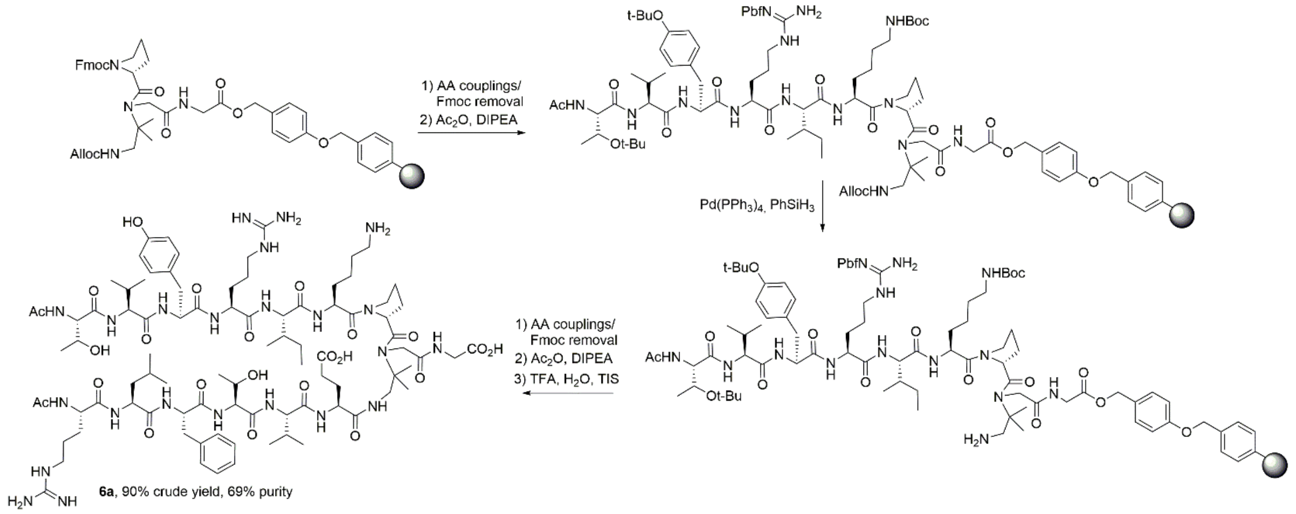

Peptide **6a** was obtained as an amorphous white solid (85 mg, 90% crude yield, 69% purity) starting from **2a** (143 mg, 0.05 mmol) according to the general procedure described above. An analytical sample (20 mg) was purified by preparative RP-HPLC for NMR, CD and HR-MS characterization. R_t = 11.1 min. HR-MS *m/z*: 631.0294 $[M+3H]^{3+}$, calcd. for $C_{88}H_{146}N_{23}O_{23}$: 631.0321.

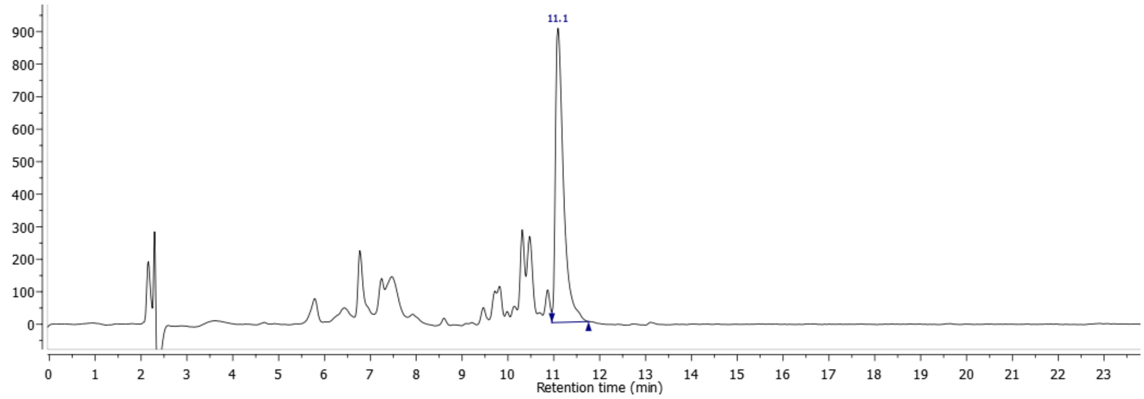

Figure S14. RP-HPLC trace of crude peptide **6a.**

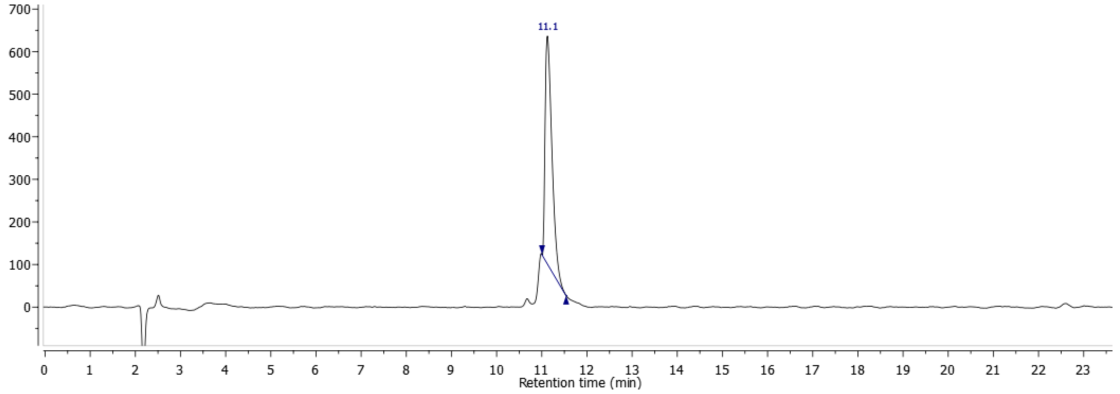

Figure S15. RP-HPLC trace of purified peptide **6a**.

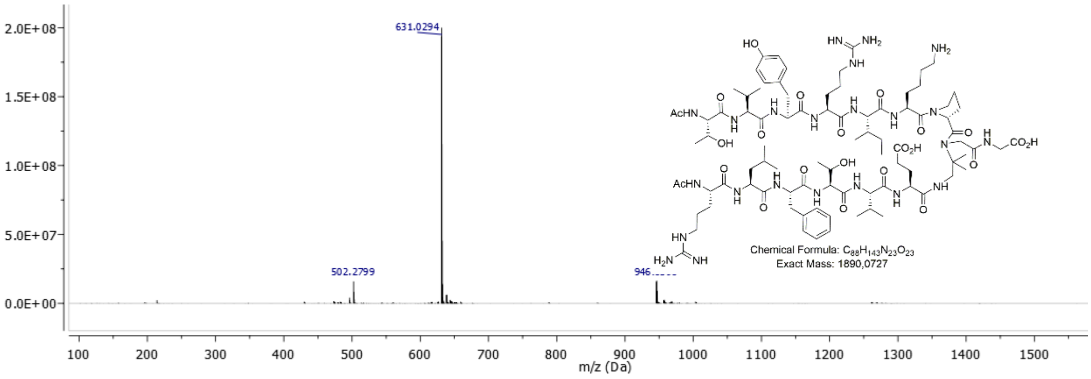

Figure S16. ESI-HRMS of peptide **6a**.

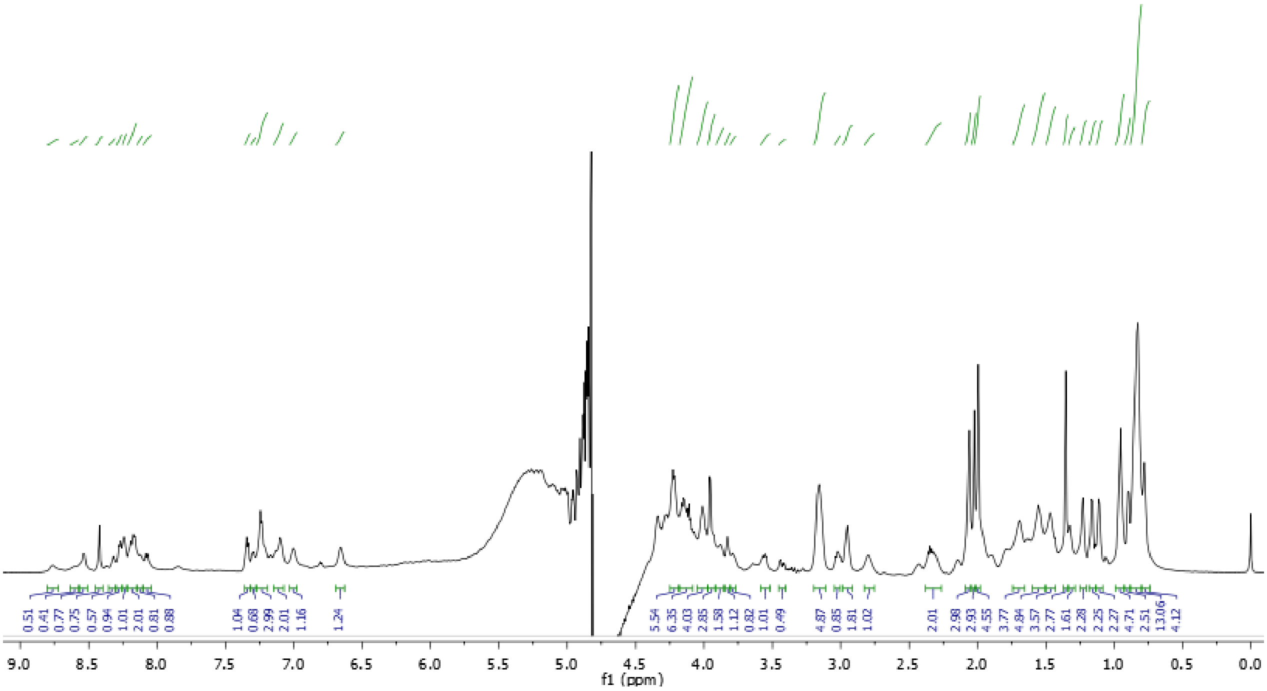

Figure S17. ^{1}H NMR spectrum (600 MHz, 9:1 H_2O:D_2O, 100 mM sodium deuteroacetate buffer, pH 3.80) of peptide **6a**.

Synthesis of peptide 6b

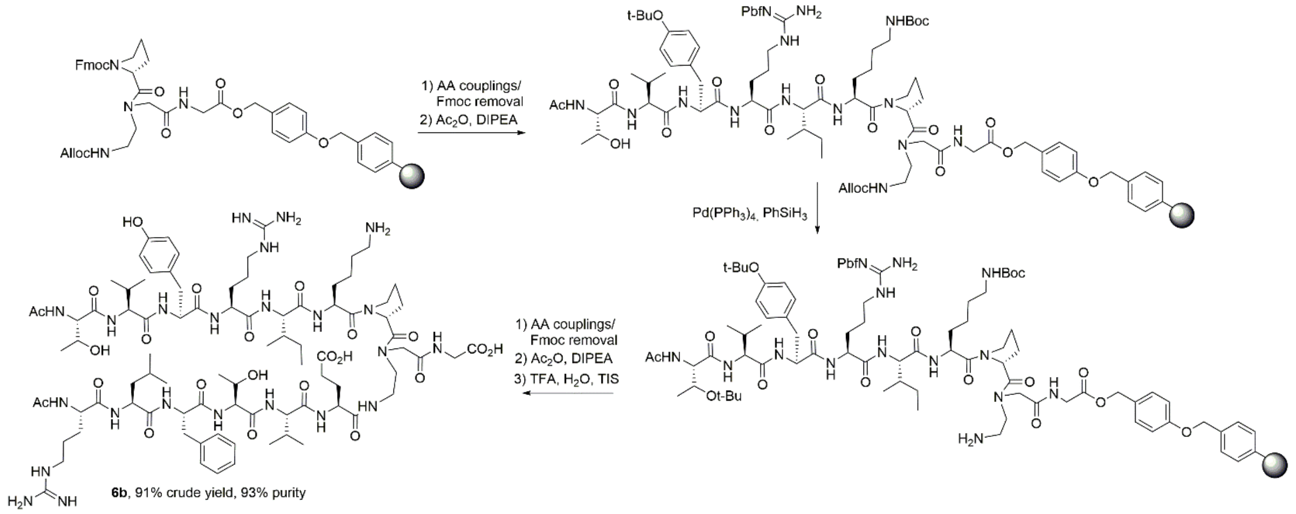

Peptide **6b** was obtained as an amorphous white solid (85 mg, 91% crude yield, 93% purity) starting from **2b** (128 mg, 0.05 mmol) according to the general procedure described above. An analytical sample (20 mg) was purified by preparative RP-HPLC for NMR and HR-MS characterization. R_t = 10.2 min. HR-MS m/z: 621.6871 $[M+3H]^{3+}$, calcd. for $C_{86}H_{142}N_{23}O_{23}$: 621.6883.

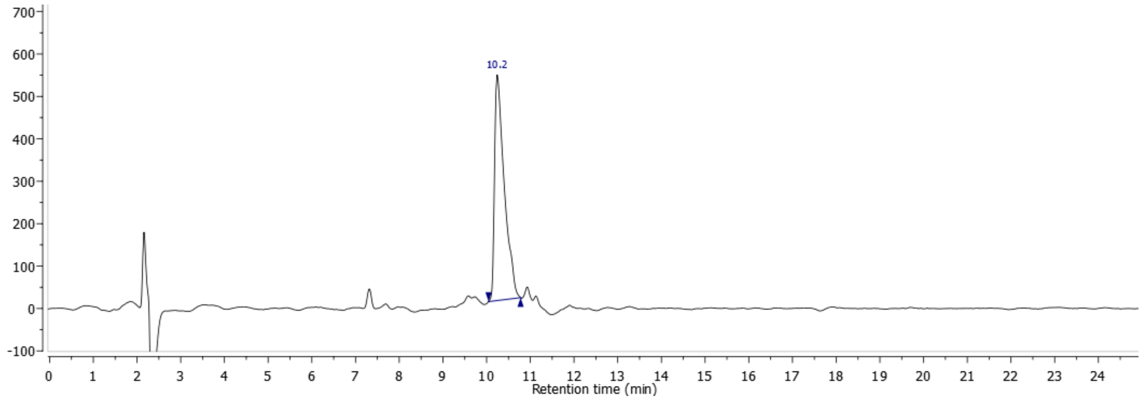

Figure S18. RP-HPLC trace of crude peptide **6b**.

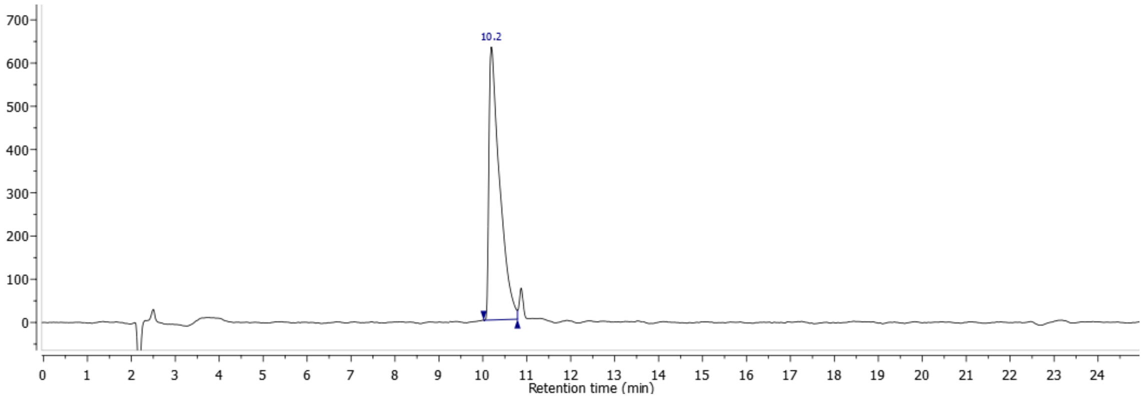

Figure S19. RP-HPLC trace of purified peptide **6b**.

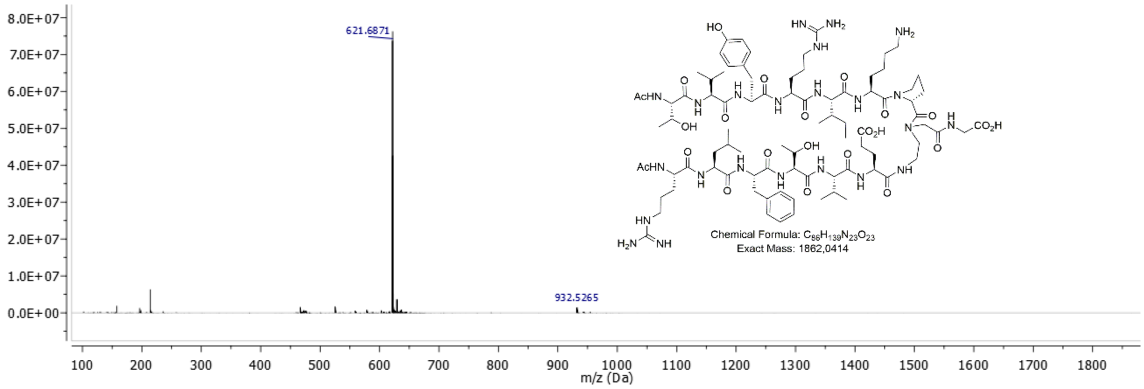

Figure S20. ESI-HRMS of peptide **6b**.

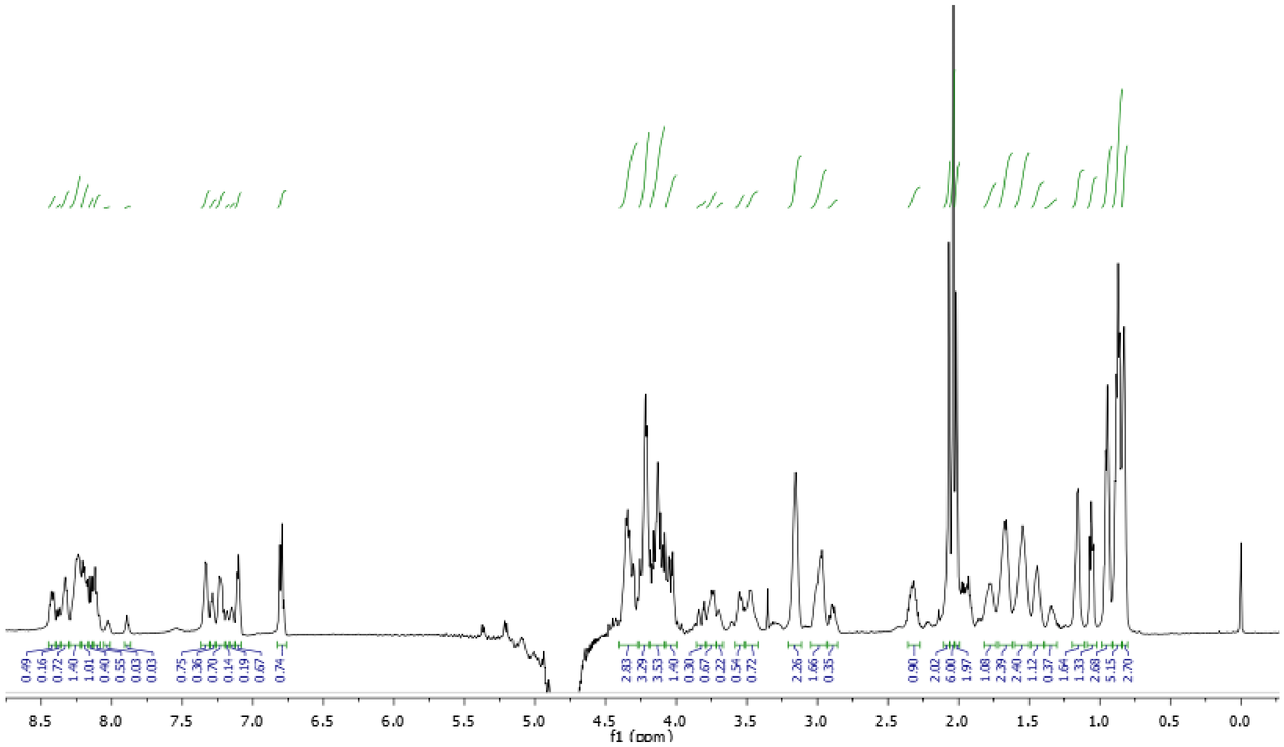

Figure S21. ^{1}H NMR spectrum (600 MHz, 9:1 H_2O:D_2O, 100 mM sodium deuteroacetate buffer, pH 3.8) of peptide **6b**.

Synthesis of peptide 7a

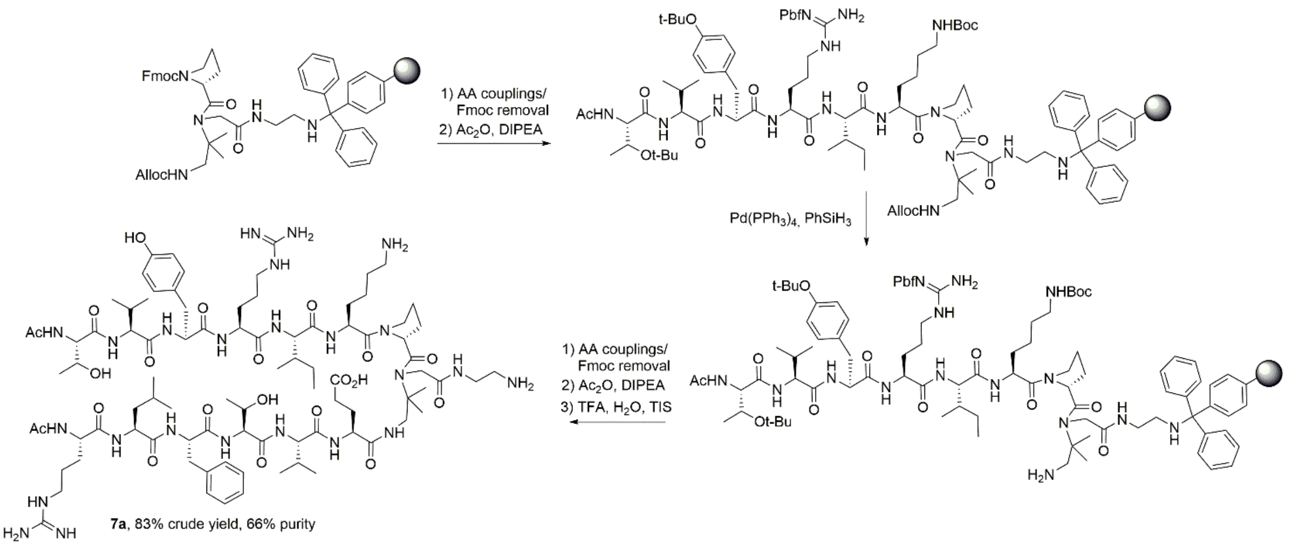

Peptide **7a** was obtained as an amorphous white solid (78 mg, 83% crude yield, 66% purity) starting from **3a** (156 mg, 0.05 mmol) according to the general procedure described above. An analytical sample (20 mg) was purified by preparative RP-HPLC for NMR, CD and HR-MS characterization. 156 mg. m= 78 mg. Crude yield: 83%, purity: 66% . R_t = 11.2 min. HR-MS m/z: 469.7831 $[M+4H]^{4+}$, calcd. for $C_{88}H_{150}N_{24}O_{21}$: 469.7852.

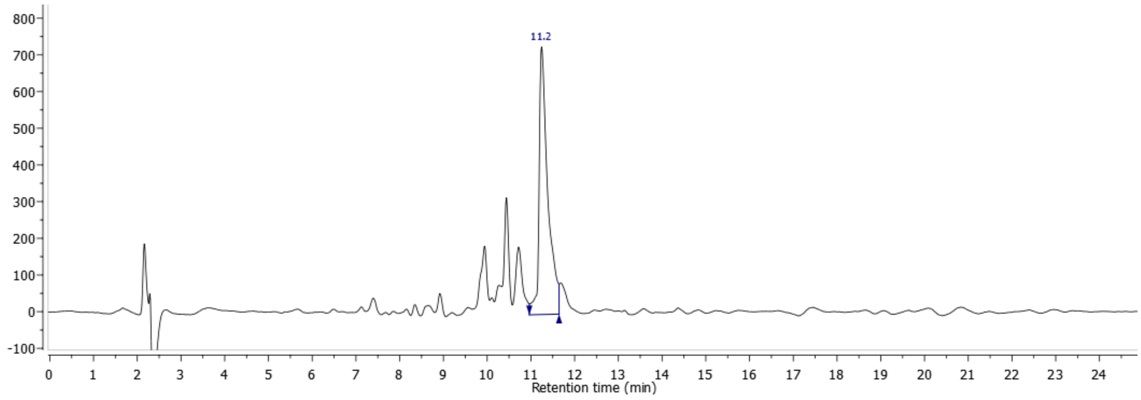

Figure S22. RP-HPLC trace of crude peptide **7a**.

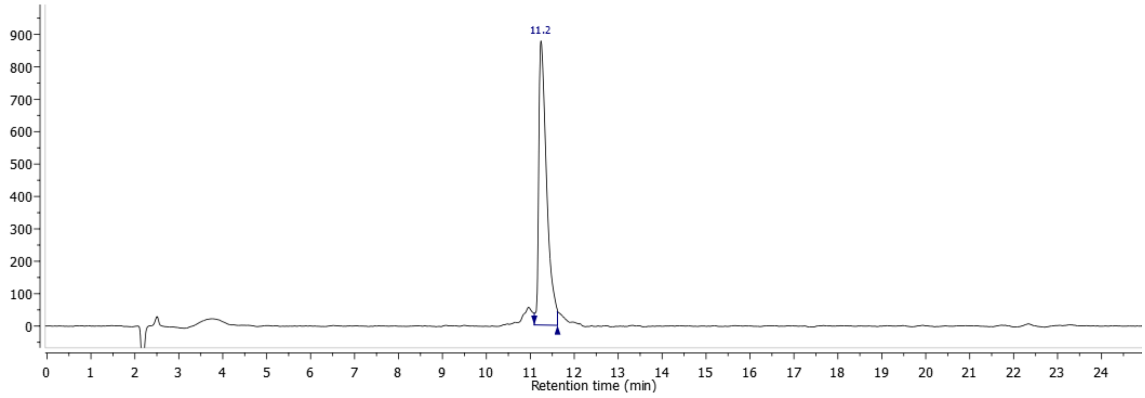

Figure S23. RP-HPLC trace of purified peptide **7a**.

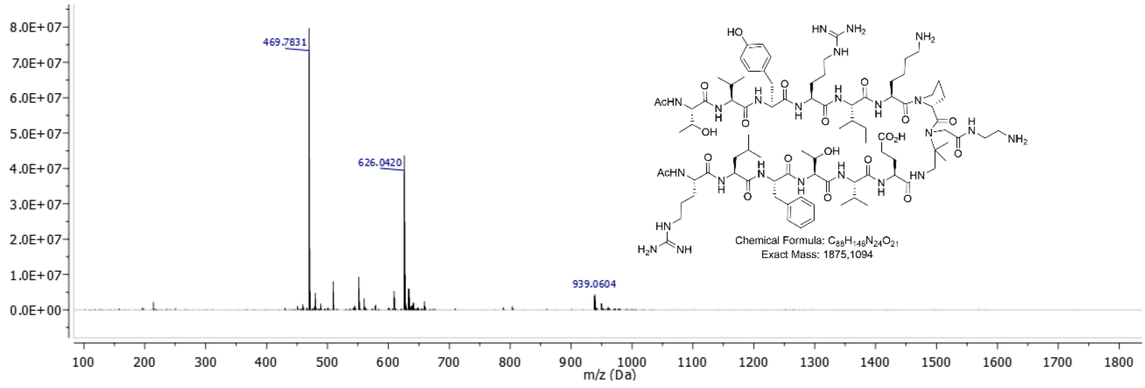

Figure S24. ESI-HRMS of peptide **7a**.

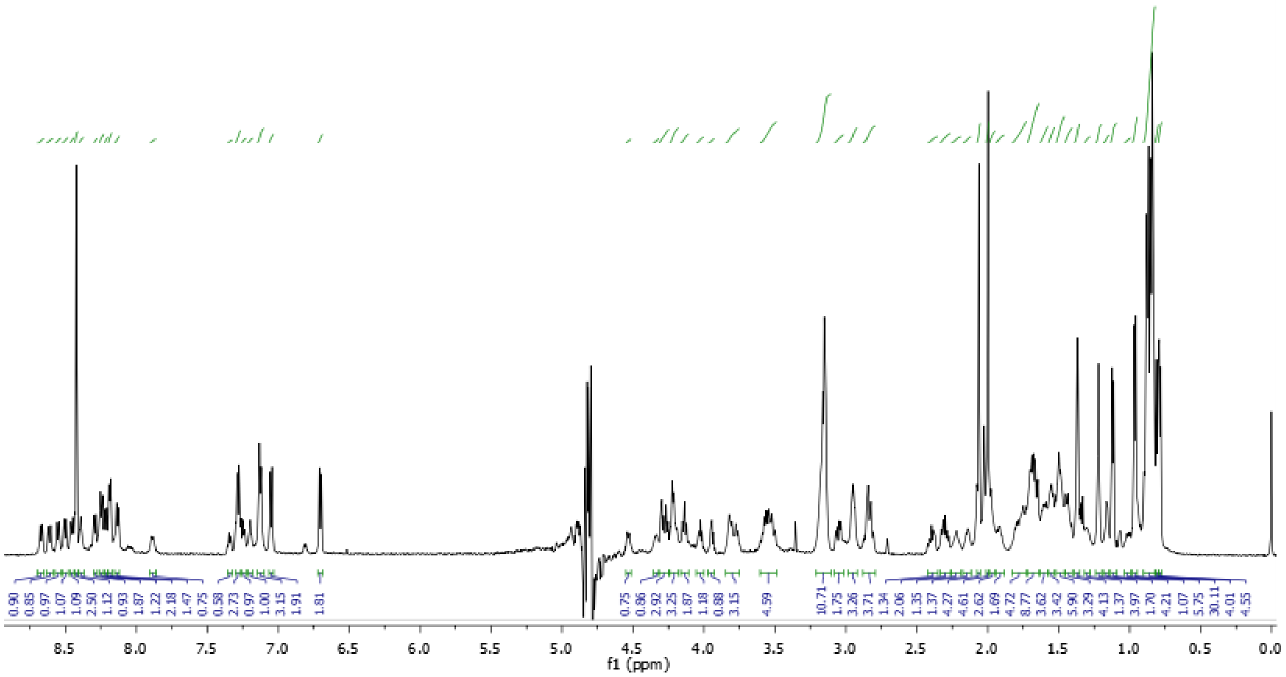

Figure S25. ^{1}H NMR spectrum (600 MHz, 9:1 H_2O:D_2O, 100 mM sodium deuteroacetate buffer, pH 3.8) of peptide **7a**.

Synthesis of peptide 7b

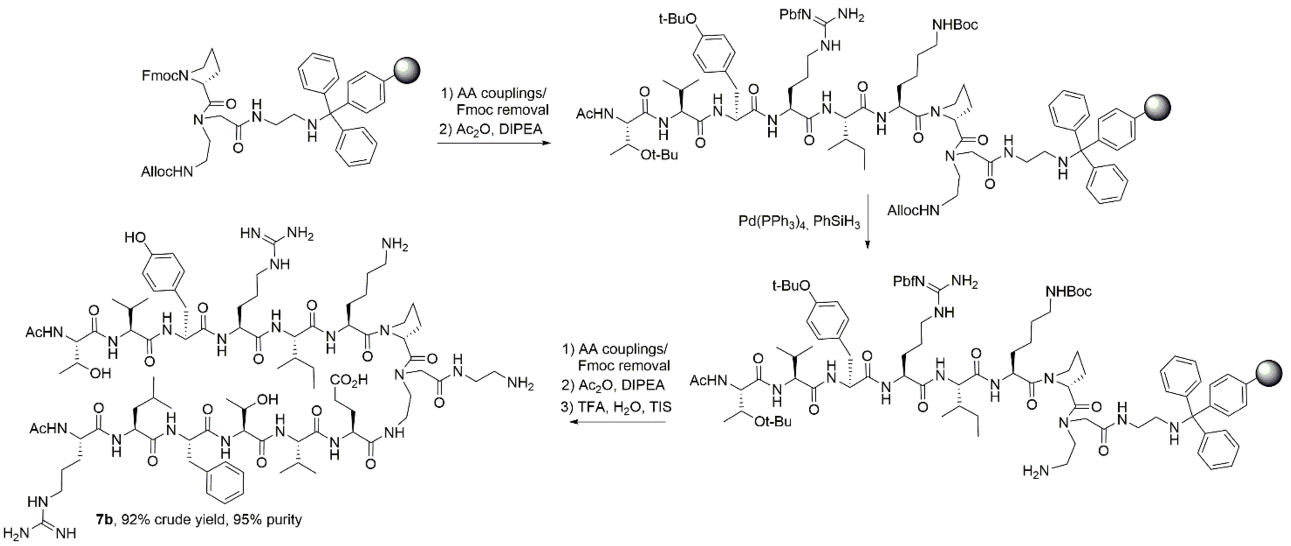

Peptide **7b** was obtained as an amorphous white solid (92 mg, 92% crude yield, 95% purity) starting from **3b** (104 mg, 0.05 mmol) according to the general procedure described above. An analytical sample (20 mg) was purified by preparative RP-HPLC for NMR, CD and HR-MS characterization. R_t = 9.1 min. HR-MS *m/z*: 662.7758 $[M+4H]^{4+}$, calcd. for $C_{86}H_{146}N_{24}O_{21}$: 462.7773.

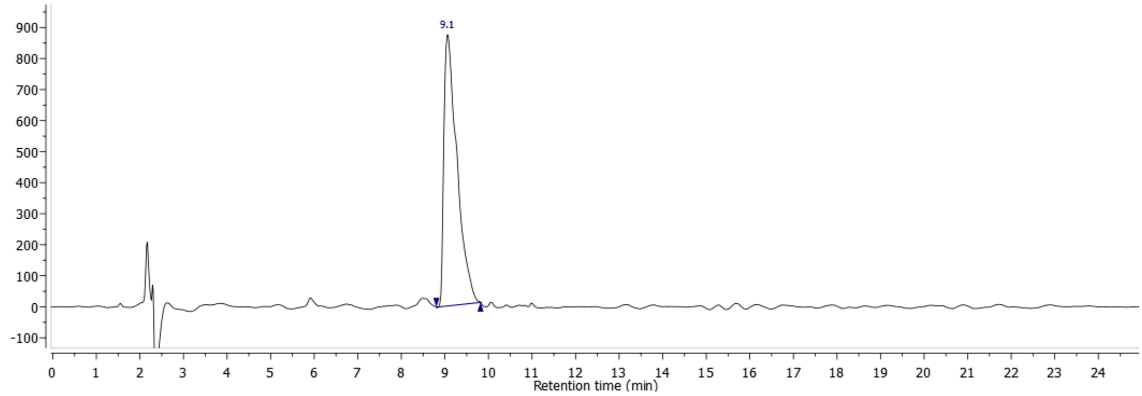

Figure S26. RP-HPLC trace of crude peptide **7b**.

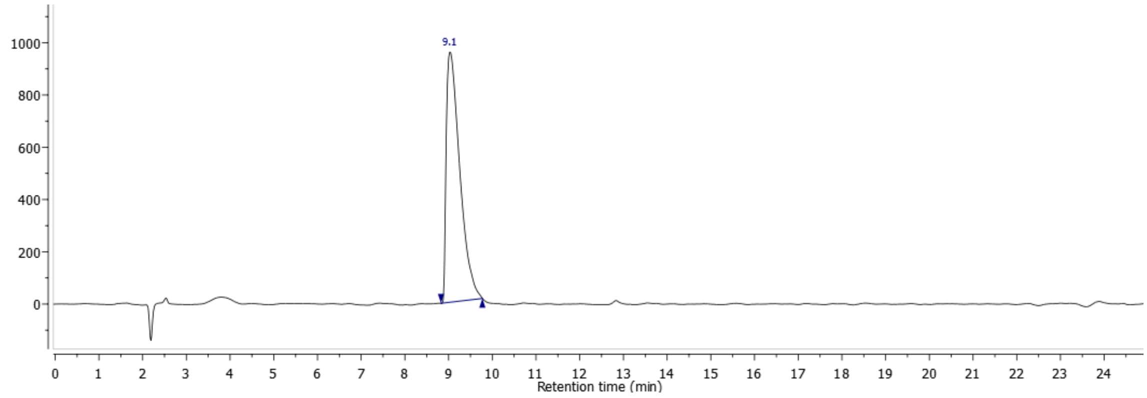

Figure S27. RP-HPLC trace of purified peptide **7b**.

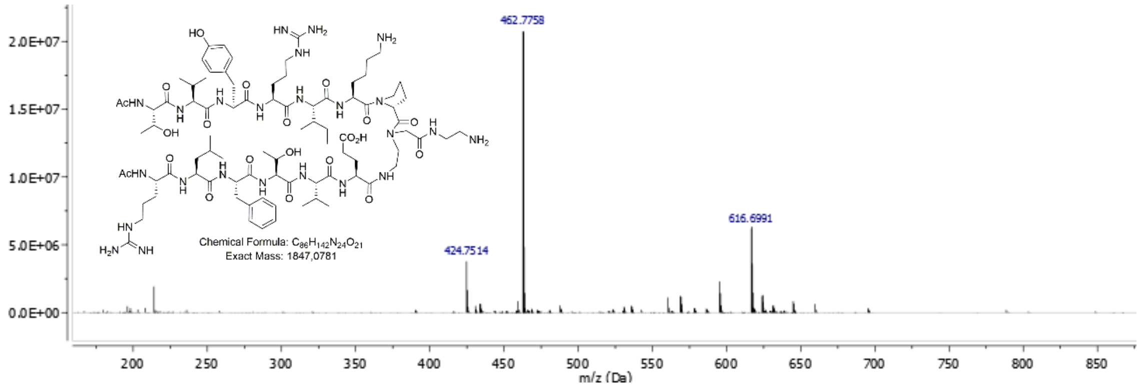

Figure S28. ESI-HRMS of peptide **7b**.

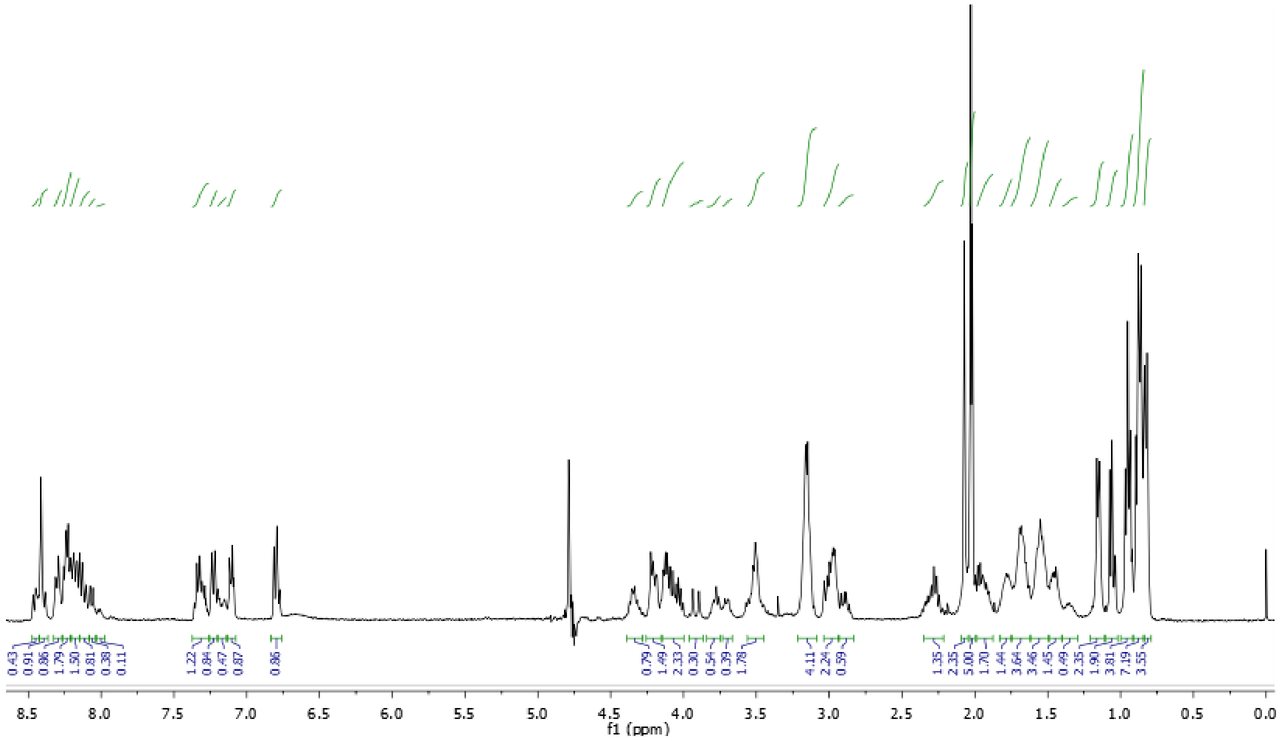

Figure S29. ^{1}H NMR spectrum (600 MHz, 9:1 H_2O:D_2O, 100 mM sodium deuteroacetate buffer, pH 3.80) of peptide **7b**.

Synthesis of peptide 8

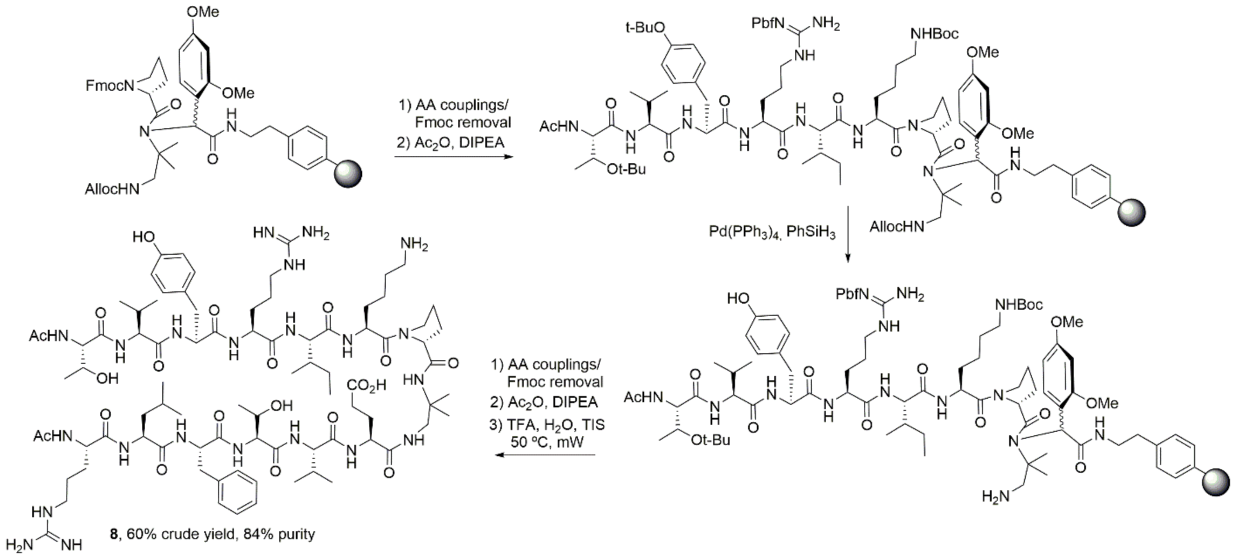

Peptide **8** was obtained as an amorphous white solid (53 mg, 60% crude yield, 84% purity) starting from **4** (180 mg, 0.05 mmol) according to the general procedure described above. This case, the cleavage was performed at 50ºC in mW for 1 h, as reported previously.[4] An analytical sample (20 mg) was purified by preparative RP-HPLC for NMR, CD and HR-MS characterization. R_t = 14.8 min. HR-MS *m/z*: 592.6900 $[M+3H]^{3+}$, calcd. for $C_{84}H_{141}N_{22}O_{20}$: 592.6898.

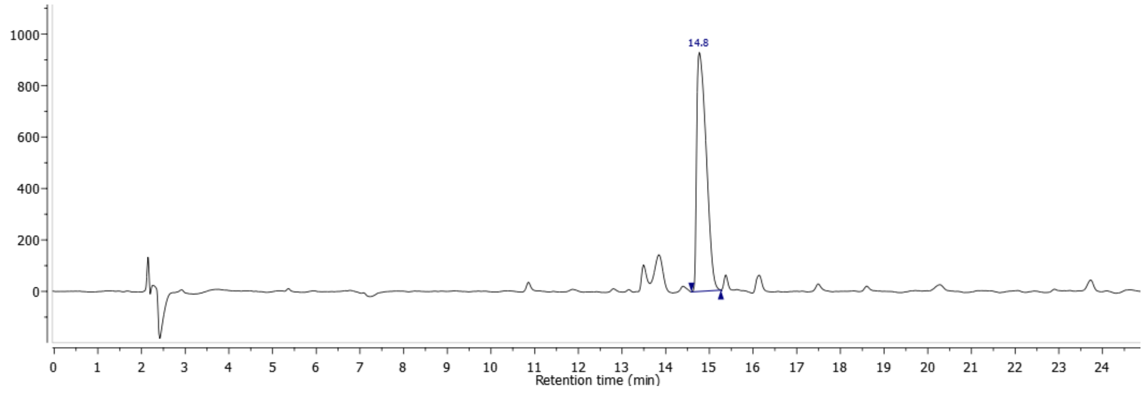

Figure S30. RP-HPLC trace of crude peptide **8.**

[4] S. Jobin, A. Méjean, S.-M. Galindo, X. Liang, S. Vézina-Dawod, E. Biron, *Org. Biomol. Chem.* **2016**, *14*, 11230.

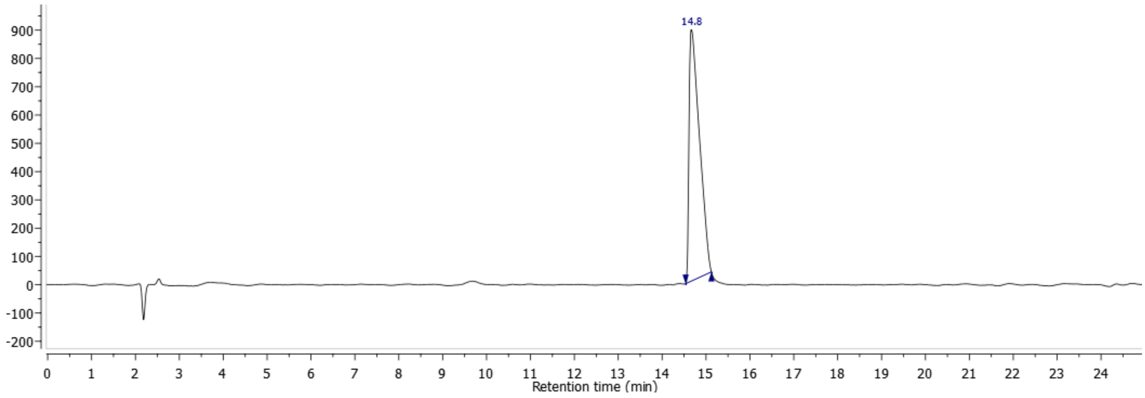

Figure S31. RP-HPLC trace of purified peptide **8.**

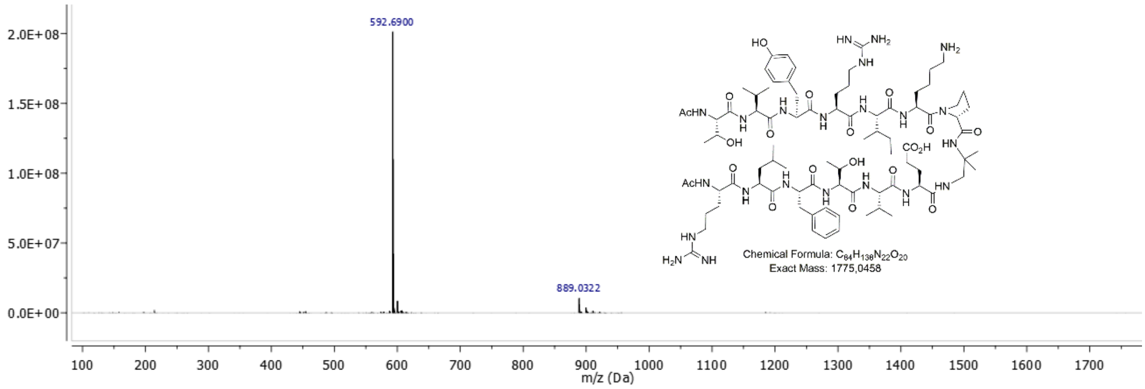

Figure S32. ESI-HRMS of peptide **8**.

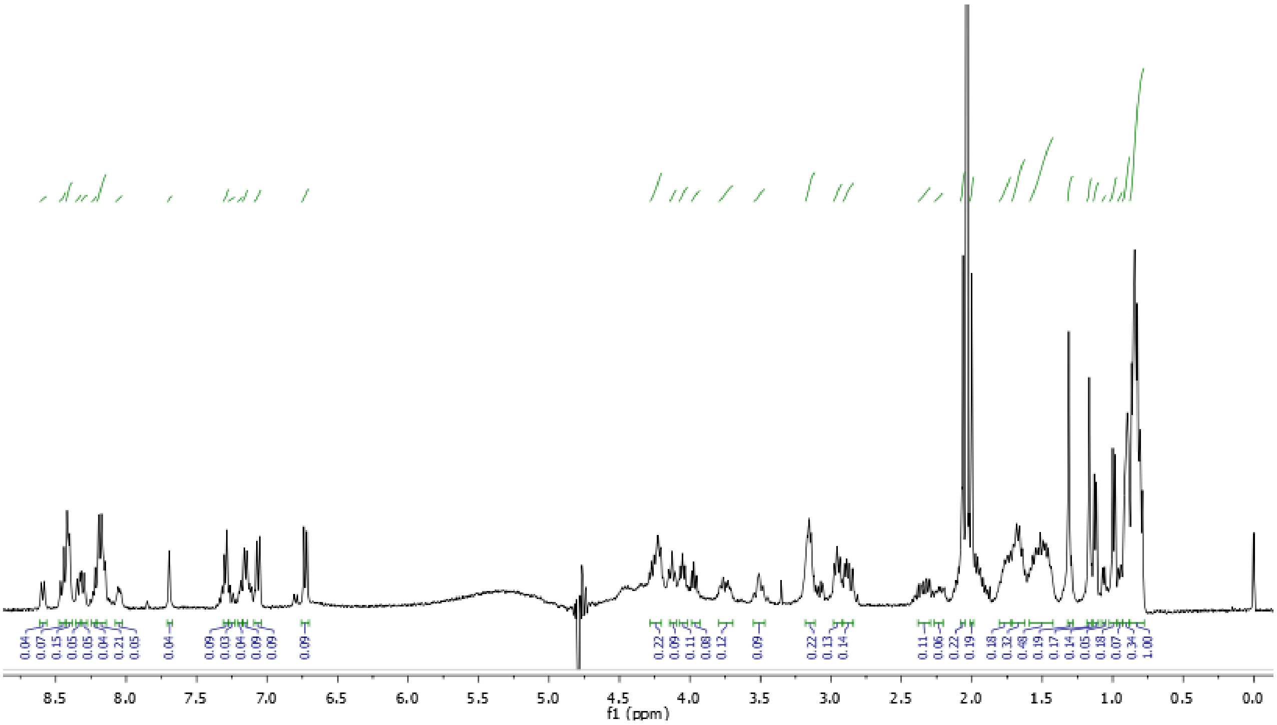

Figure S33. ^{1}H NMR spectrum (600 MHz, 9:1 H_2O:D_2O, 100 mM sodium deuteroacetate buffer, pH 3.8) of peptide **8**.

Synthesis of peptide 12

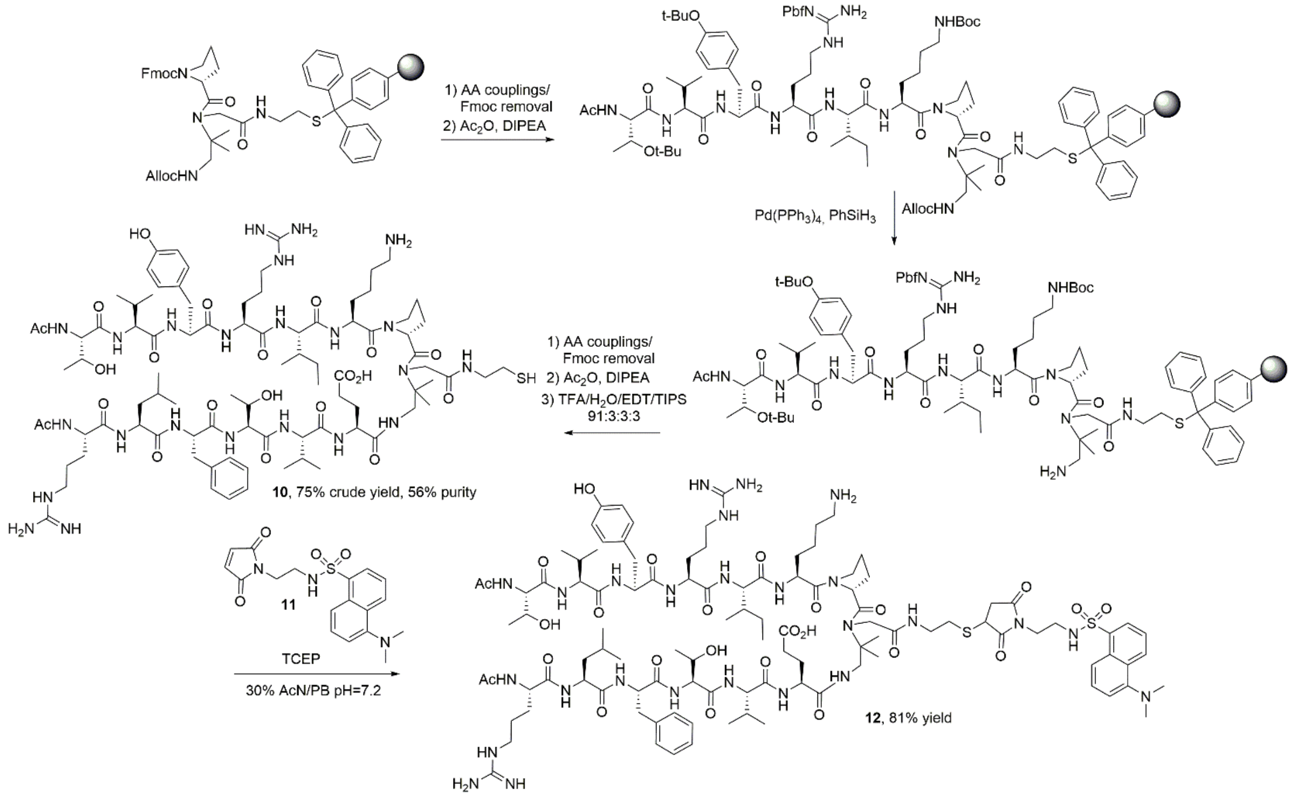

Peptide **10** was obtained as an amorphous white solid after lyophilization (83 mg, 75% crude yield, 56% purity) starting from the resin-linked peptide **9** (192 mg, 0.05 mmol) and following the general procedure described above. 40 mg of the crude peptide was purified by preparative RP-HPLC and characterized by NMR, CD and HR-MS. R_t = 11.8 min. HR-MS *m/z*: 631.7015 $[M+3H]^{3+}$, calcd. for $C_{88}H_{148}N_{23}O_{21}S$: 631.6980. In 15 mL of a mixture of 0.1 M buffer phosphate/acetonitrile 2:1, the purified peptide **10** (15 mg, 7.9 µmol), *N*-[2-(dansylamino)ethyl]maleimide (**11**, 4.3 mg, 15.8 µmol) and TCEP (3.9 mg, 15.8 µmol) were stirred at room temperature for 6 h. Then, the crude mixture was directly purified by preparative RP-HPLC to afford peptide **12** (12.7 mg, 81 %) as an amorphous white solid. R_t = 13.9 min. HR-MS *m/z*: 756.0783 $[M+3H]^{3+}$, calcd. for $C_{106}H_{167}N_{26}O_{25}S_2$: 756.0679.

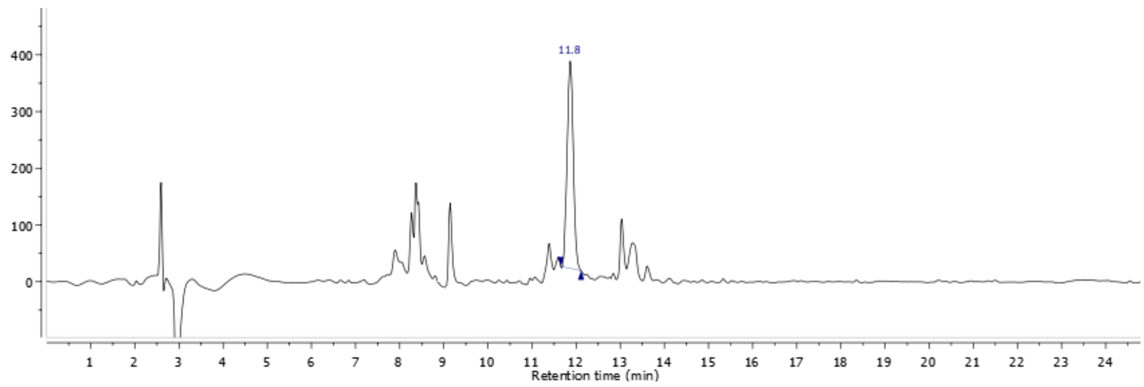

Figure S34. RP-HPLC trace of crude peptide **10**.

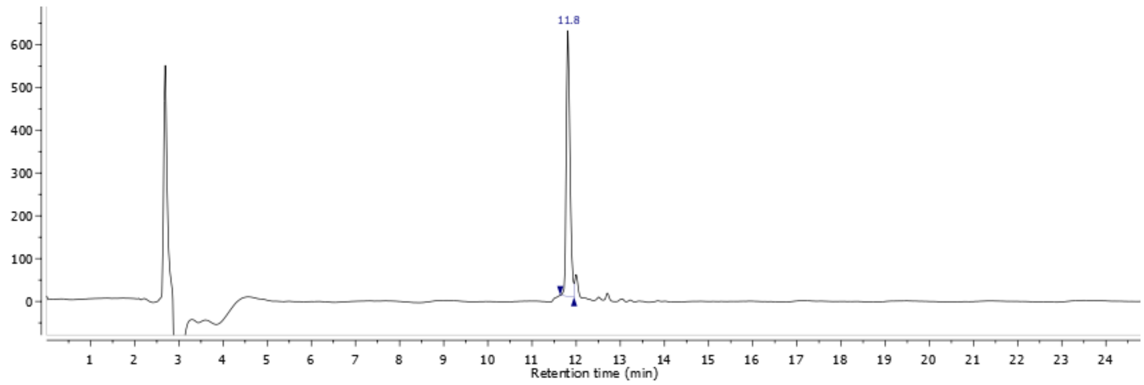

Figure S35. RP-HPLC trace of purified peptide **10**.

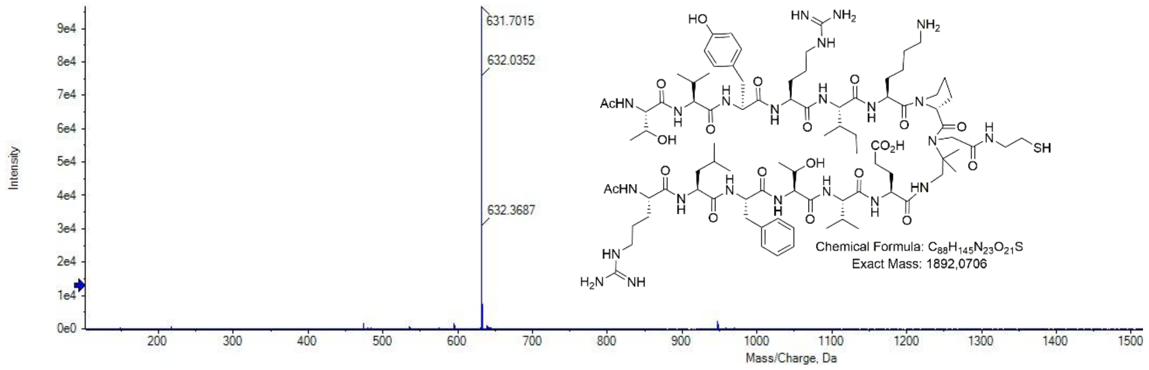

Figure S36. ESI-HRMS of peptide **10**.

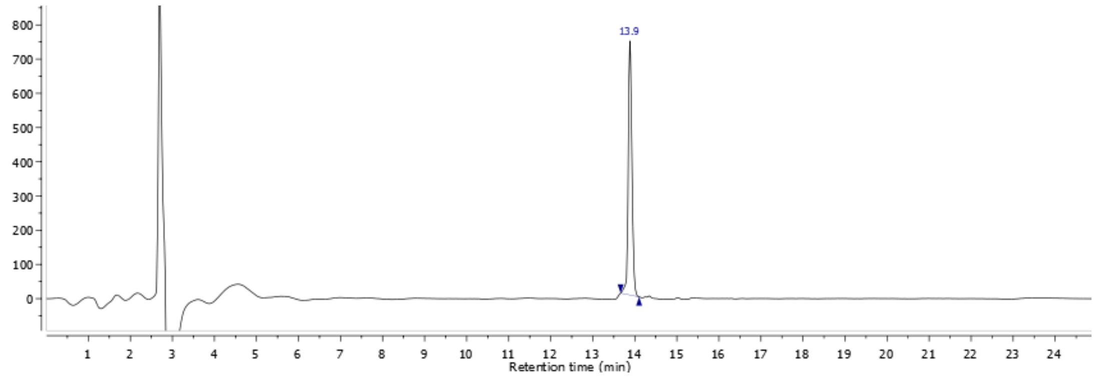

Figure S37. RP-HPLC trace of purified peptide **12**.

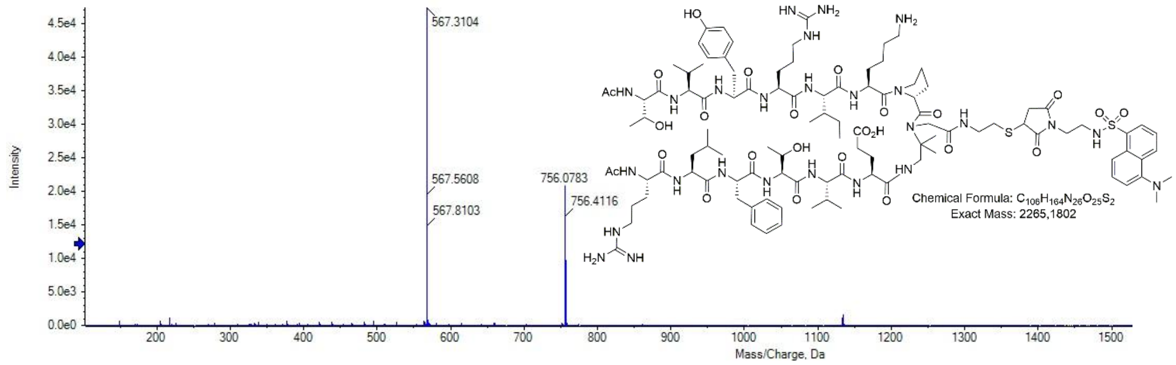

Figure S38. ESI-HRMS of peptide **12**.

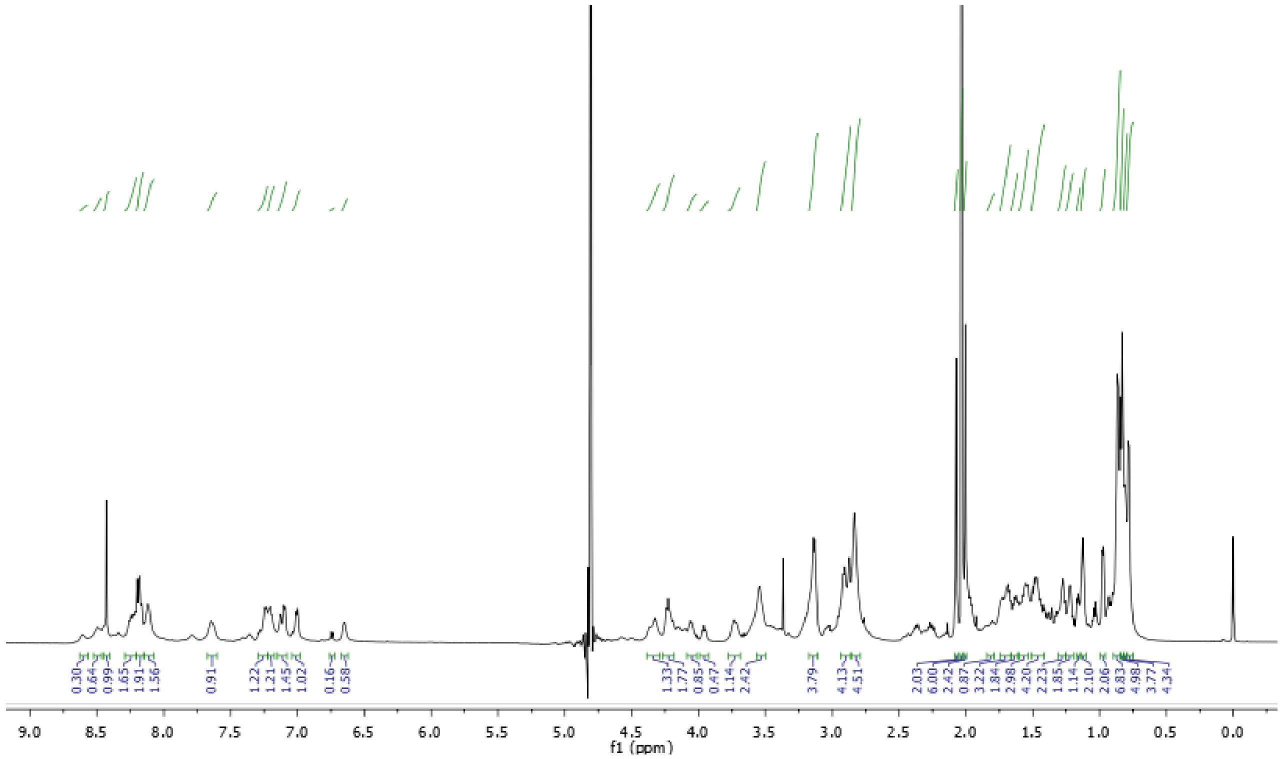

Figure S39. ^{1}H NMR spectrum (600 MHz, 9:1 H_2O:D_2O, 100 mM sodium deuteroacetate buffer, pH 3.8) of peptide **12**.

Synthesis of *N*-[2-(dansylamino)ethyl]maleimide 11

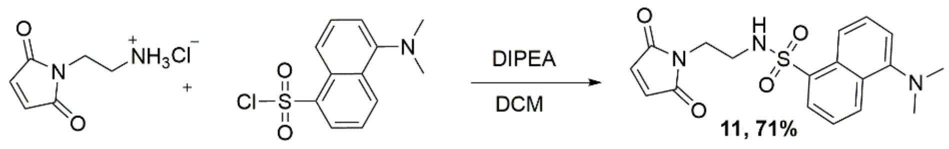

2-Maleimidoethylamine hydrochloride (200 mg, 1,14 mmol) and dansyl chloride (307 mg, 1,14 mmol) are mixed with DIPEA (400 µL, 2.28 mmol) in DCM (10 mL) at room temperature and left stirring overnight. Then, the reaction mixture is diluted with chloroform (30 mL), washed with saturated $NaHCO_3$ solution (2×10 mL) and brine (2×10 mL), dried over anh. Na_2SO_4, and

concentrated under reduced pressure to dryness. The crude product is purified by column chromatography (DCM/EtOAc 3:1) to afford pure *N*-[2-(dansylamino)ethyl]maleimide **11** (298 mg, 71%) as a bright yellow solid. ^{1}H NMR (400 MHz, Chloroform-*d*) δ = 8.52 (dt, *J* = 8.6, 1.1 Hz, 2H), 8.22 – 8.15 (m, 2H), 7.53 (ddd, *J* = 12.5, 8.5, 7.4 Hz, 2H), 7.16 (dd, *J* = 7.6, 0.8 Hz, 2H), 6.35 (s, 2H), 5.08 (t, *J* = 6.1 Hz, 1H), 3.53 (t, *J* = 6.0 Hz, 2H), 3.17 (q, *J* = 5.9 Hz, 2H), 2.88 (s, 6H). ^{13}C NMR (101 MHz, cdcl$_3$) δ = 170.8, 170.4, 152.0, 134.4, 134.1, 133.3, 130.55, 130.0, 129.9, 129.3, 128.5, 123.3, 118.7, 115.1, 45.4, 41.7, 39.6, 36.9, 36.7.

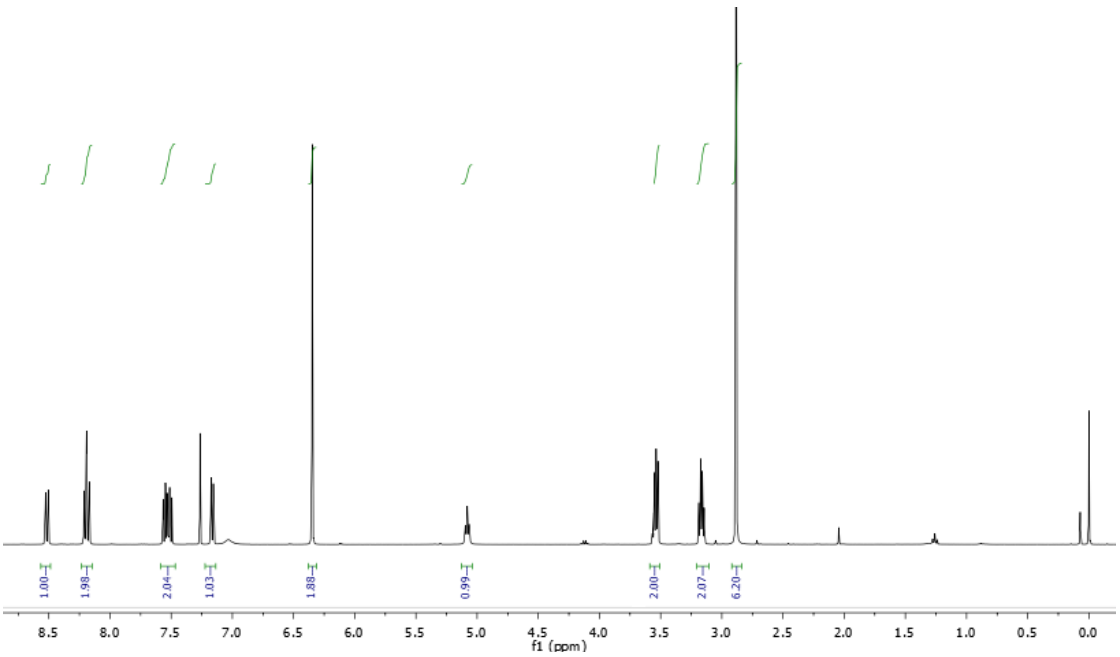

Figure S40. ^{1}H NMR spectrum (400 MHz, CDCl$_3$) of *N*-[2-(dansylamino)ethyl]maleimide **11**.

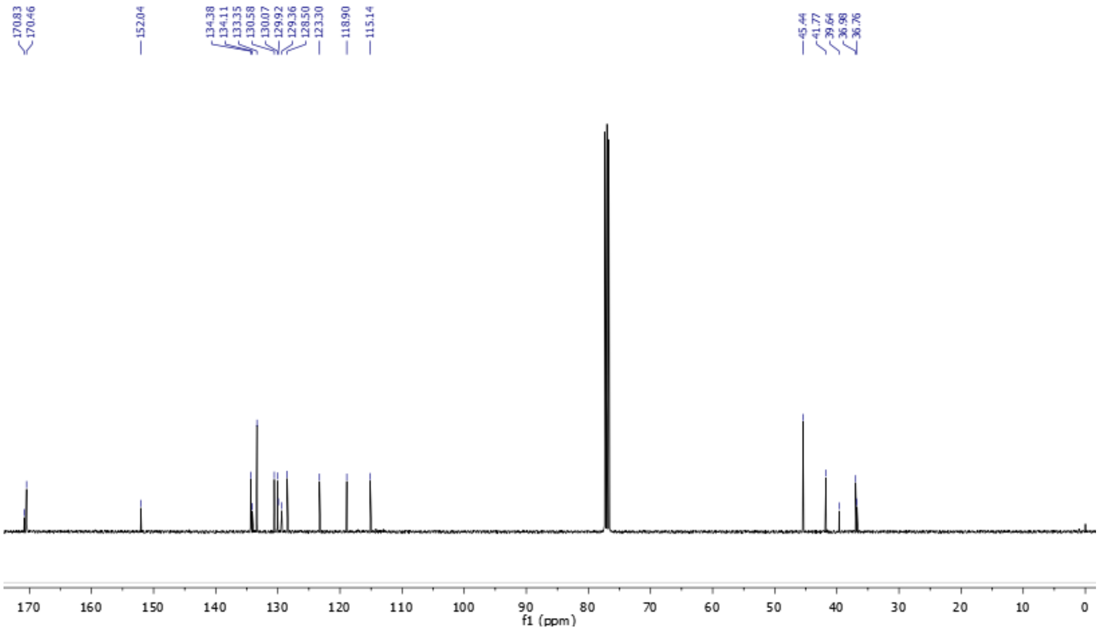

Figure S41. ^{13}C NMR spectrum (400 MHz, CDCl$_3$) of *N*-[2-(dansylamino)ethyl]maleimide **11**.

Circular dichroism analysis

Circular dichroism spectra were collected over the wavelength range 185-260 nm with a Jasco J-815 spectropolarimeter equipped with a temperature controller at 25 ºC. 100 µM peptide solutions were prepared as follow: lyophilized peptides were weighted and dissolved in 10 mM sodium acetate buffer (AcB), pH 3.8 or in mixtures with TFE. All spectra were obtained using standard measurement parameters: 50 nm/sec speed, 16 accumulations, 1 mm path length and converted to molar ellipticity after background subtraction and smoothing.

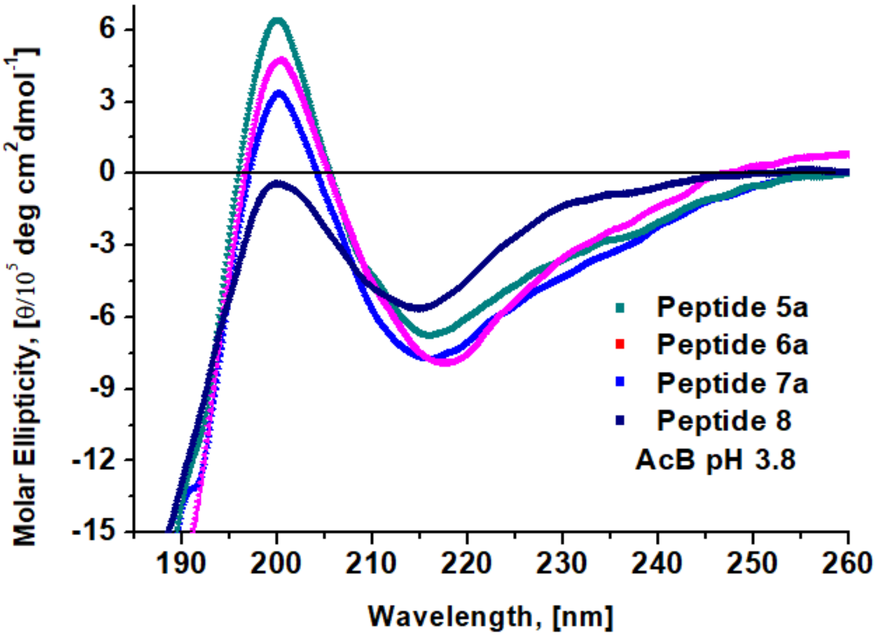

Figure S42. CD spectra of peptides **5a**, **6a**, **7a** and **8** in AcB pH 3.8.

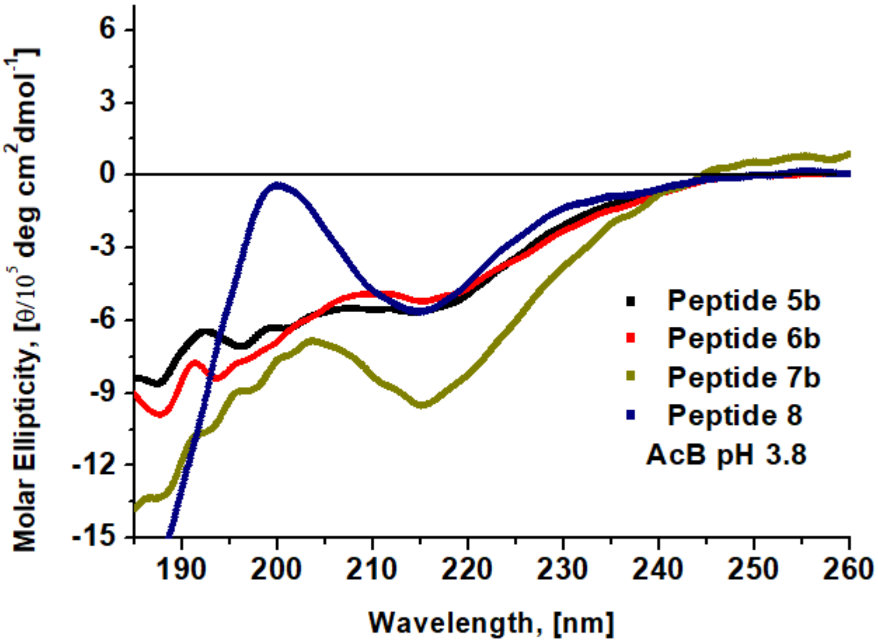

Figure S43. CD spectra of peptides **5b**, **6a**, **7b** and **8** in AcB pH 3.8.

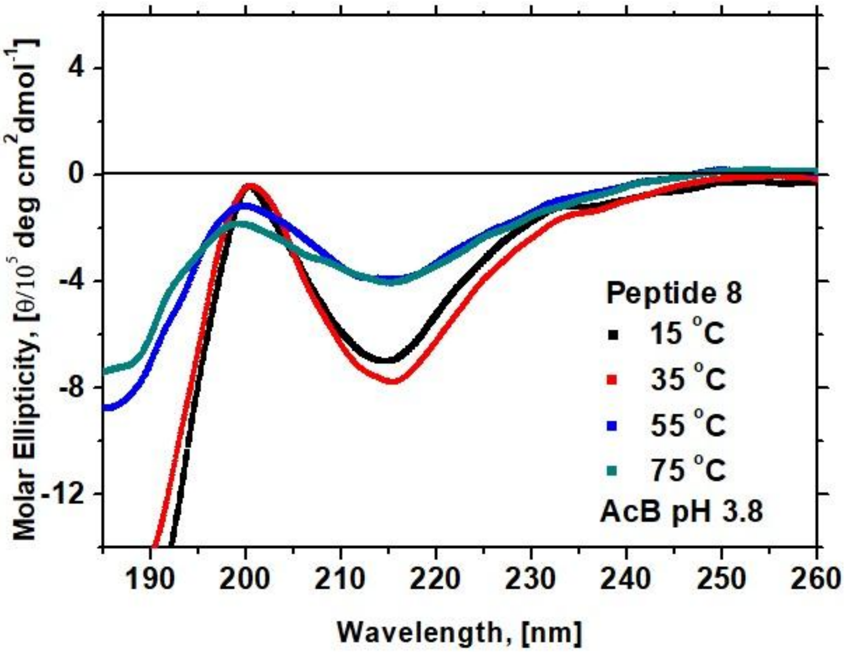

Figure S44. CD spectra of peptide **8** in AcB pH 3.8 at different temperatures.

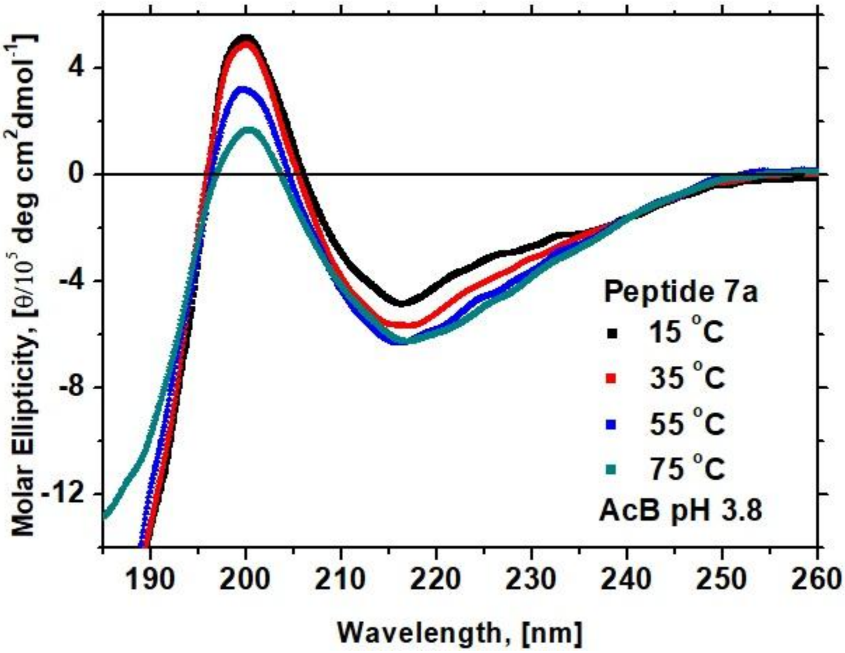

Figure S45. CD spectra of peptide **7a** in AcB pH 3.8 at different temperatures.

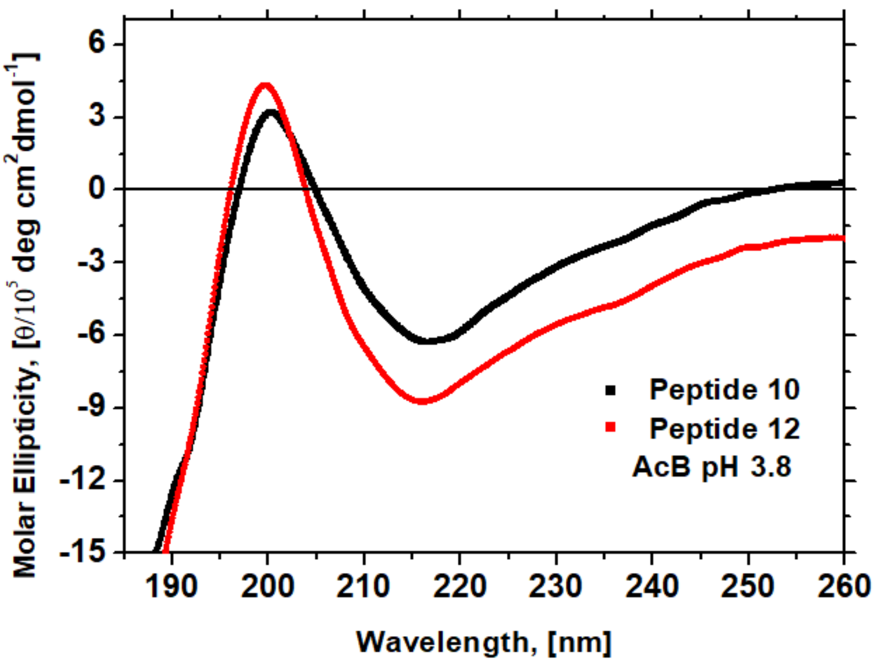

Figure S46. CD spectra of peptides **10** and **1**2 in AcB pH 3.8.

NMR analysis and simulated annealing

Sample preparation and experiments

NMR samples were prepared from all lyophilized peptides by dissolving them in 9:1 H_2O/D_2O (0.03% of 2,2-dimethyl-2-silapentane-5-sulfonate, DSS), 100 mM deuterium acetate buffer pH = 3.8 at 2.5 mM concentration. NMR spectra were acquired at 298 K on a Varian VNMRS 600 spectrometer operating at a proton NMR frequency of 599.83 MHz using a 5-mm inverse detection cryoprobe and using standard CHEMPACK 8.1 pulse sequences (water_ES, gDQCOSY, zTOCSY_ES, ROESYAD_ES, gHSQCAD, gHMBCAD) implemented in Varian VNMRJ 4.2A spectrometer software. For ^{1}H, TOCSY (mixing time 80 msec) as well as ROESY (mixing time 0.2 s) measurements the water signal was suppressed by excitation sculpting. COSY, HSQC and HMBC spectra were recorded with water suppression by presaturation. The HSQC experiment was optimized for $^{1}J_{CH}$ = 146 Hz and the HMBC experiment was optimized for a long-range coupling of 8 Hz.

Structure calculations

Peptide **7a** was chosen for structural elucidation by NMR. For this purpose, resonances were fully assigned by analysis TOCSY COSY, HSQC, HMBC, and ROESY spectra. Cross-peaks in ROESY spectra were assigned and integrated in SparkyNMR 3.113.[5] Distance constraints were derived from the intensities of ROE cross-peaks from the ROESY spectrum and grouped as: strong (2.8 Å upper limit), medium (3.5 Å upper limit) and weak (5.0 Å upper limit). A total of 86 distance constraints resulted from analysis of ROE intensities. A more detailed classification is given in the tables 2 and 3.

Backbone dihedral angle restraints were inferred from $^{3}J_{NHH\alpha}$ coupling constants in the ^{1}H spectrum at 283 K, φ was restrained to -120 ± 30° for $^{3}J_{NHH\alpha} \geq 8$ Hz. NMR structure determination was performed in XPLOR-NIH 2.49 package[6] through simulated annealing regularization and refinement in torsion angle space, using experimental data as inter-proton distances and dihedral angles restraints. Simulations were based on examples scripts distributed in the "/eginput" folder within Xplor-NIH package. The calculations were performed using the standard force field parameter set (PARALLHDG.PRO) and topology file (TOPALLHDG.top) with in-house modifications to generate the β-sheet inductor and the cis-trans isomerization of the DADME-PRO

[5] W. Lee, M. Tonelli, J. L. Markley, *Bioinformatics (Oxford, England)*, **2014**, *31*, 1325.
[6] (a) C. D. Schwieters, J. J. Kuszewski, N. Tjandra, G. M. Clore, *J. Magn. Res.* **2003**, *160*, 66. (b) C. D. Schwieters, J. J. Kuszewski, N. Tjandra, G. M. Clore, *Progr. NMR Spectroscopy* **2006**, *48*, 47.

amide bond. Structures were visualized and analyzed using VMD-XPLOR 1.7,[7] Chimera 1.13,[8] PyMol 2.0.[9]

Table 1. Proton resonances for peptide **7a** relative to DSS.

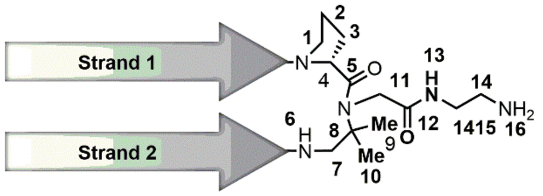

Peptide 7a

Atom	δ_H (^3J)/δ_C	Atom	δ_H/δ_C
**1**	3.77-3.82/51.33	**9**	1.22/28.5
**2**	2.14-1.98/27.7	**10**	1.35/25.9
**3**	1.93-2.21/31.9	**11**	4.22/(n/d)
**4**	4.63/(n/d)	**12**	174.9
**5**	177.8	**13**	8.39
**6**	7.89 (9.4)	**14**	3.56/40.0
**7**	3.52-3.81/48.7	**15**	3.15/42.0
**8**	64.4	**16**	7.13 (7.4)/7.28 (7.4)

	Residue	HN($^3J_{HNHa}$)	HA/CA	HB/CB	CO	others
Strand 1	**Lys[1]**	8.67 (9.1)	4.88/66.9	1.76-1.61/34.2	177.2	3.18/39.8 ε; 7.20 NH_2
	**Ile[2]**	8.46 (8.3)	4.54/56.2	1.71/39.1	176.0	1.01/25.0 γCH_2; 0.81/17.7 γCH_3; 0.84/24.9 δ
	**Arg[3]**	8.55 (8.6)	4.53/55.4	1.74-1.80/32.2	174.6	1.60-1.55/27.3 γ; 3.18/43.7 δ
	**Tyr[4]**	8.29 (8.3)	4.95/57.4	2.84-3.02/39.8	175.7	7.05/133.3 δ;7.11/133.2 ε; 157.4 (OH)
	**Val[5]**	8.25 (8.7)	4.14/62.1	1.97/33.4	174.8	0.85/21.0 γ
	**Thr[6]**	8.19 (n/d)	4.30/62.7	3.95/70.1	174.5	0.97/21.9 γ
	**Ac**	-	2.06/24.7	-	177.3	-
Strand 2	**Glu[7]**	8.42 (n/d)	4.63/56.6	2.39-2.30/36.1	176.7	2.04/30.5 γ
	**Val[8]**	8.61 (9.1)	4.26/61.2	2.00/34.3	174.8	0.89/24.9 γ
	**Thr[9]**	8.22 (8.7)	4.02/70.3	4.85/ 59.0	174.0	1.12/22.1 γ
	**Phe[10]**	8.50 (8.3)	4.71/52.9	3.06-2.83/40.6	175.1	7.13/132.0 γ
	**Leu[11]**	8.13 (7.6)	4.53/54.9	1.48/27.3	176.7	0.79/24.2-0.84/24.9 γ
	**Arg[12]**	8.18 (n/d)	4.22/56.4	1.69/51.2	175.9	1.56-1.61/27.4 γ; 3.15/43.6 δ; 127.8 ζ; 7.12-6.8 (NH)
	**Ac**	-	2.00/24.6	-	177.0	-

[7] G. M. Clore, *J. Magn. Res.* **2001**, *149*, 239.
[8] E. F. Pettersen, T. D. Goddard, C. C. Huang, G. S. Couch, D. M. Greenblatt, E. C. Meng, T. E. Ferrin, *J Comput Chem.* **2004,** *25*, 1605.
[9] W. L. DeLano, *PyMOL. DeLano Scientific, San Carlos, CA, 700*, **2002**.

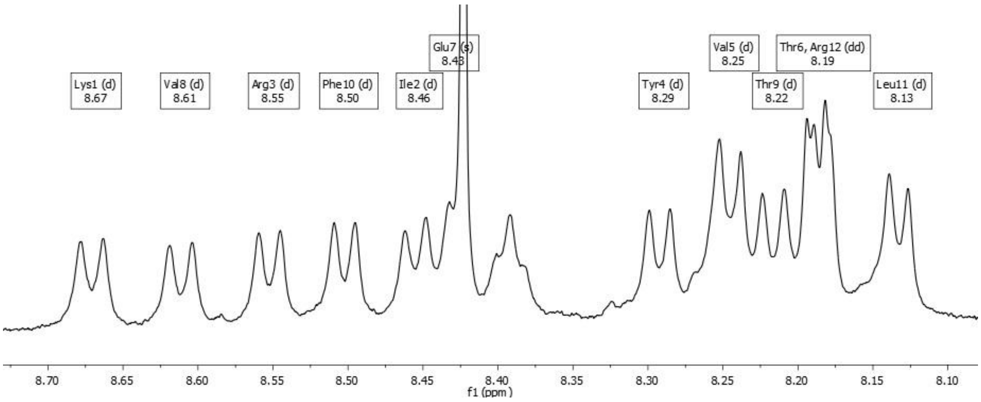

Figure 47. NMR NH resonances of peptide **7a**.

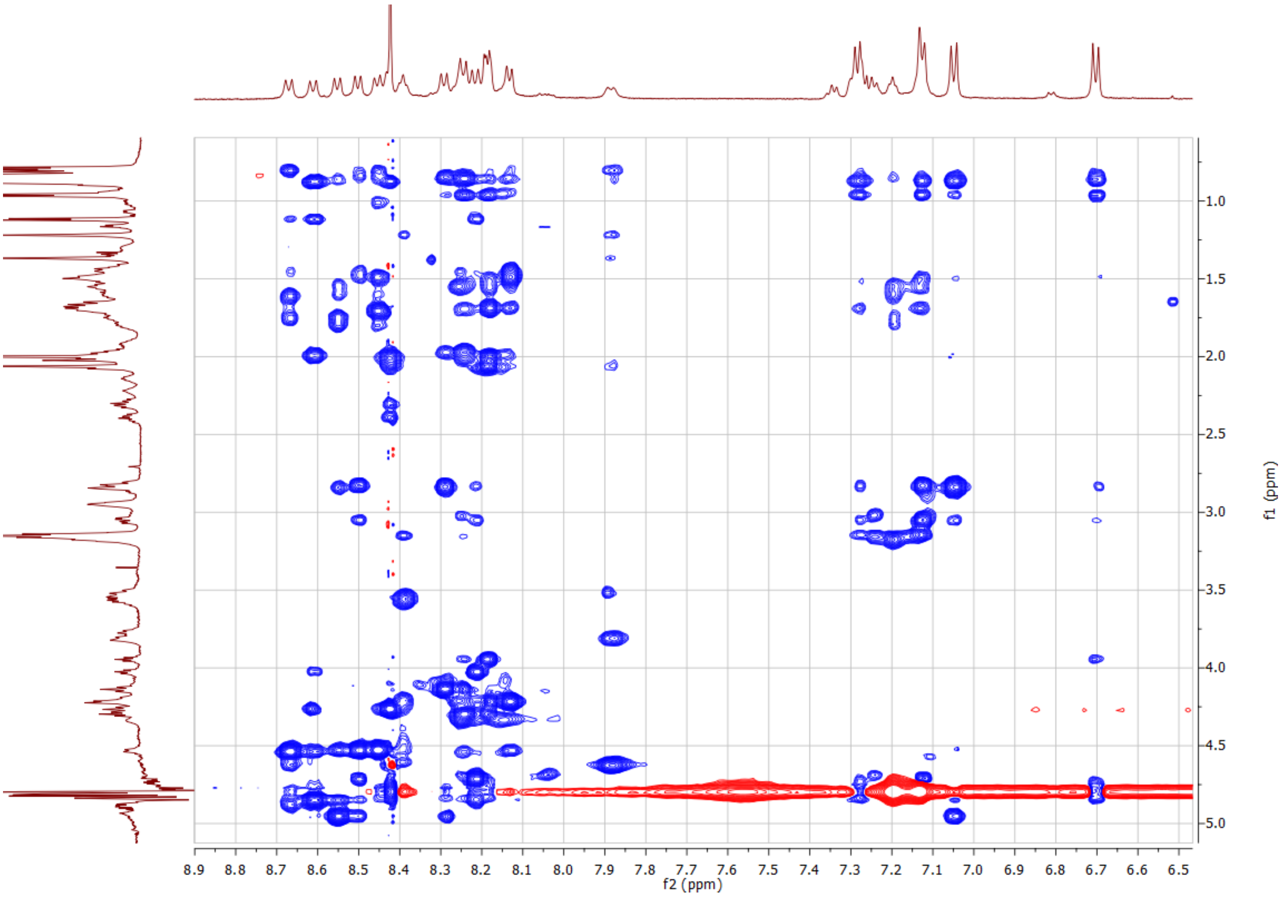

Figure 48. ROESY spectrum of peptide **7a** in aqueous solution at 283 K.

Table 2. Observed NOE contacts characteristic of parallel β-sheet structure for peptide **7a** in aqueous solution at 283 K.

NOE intensity	Total	Intra-residual	Sequential
**Strong**	53	32	15
**Medium**	26	16	9
**Weak**	7	2	3

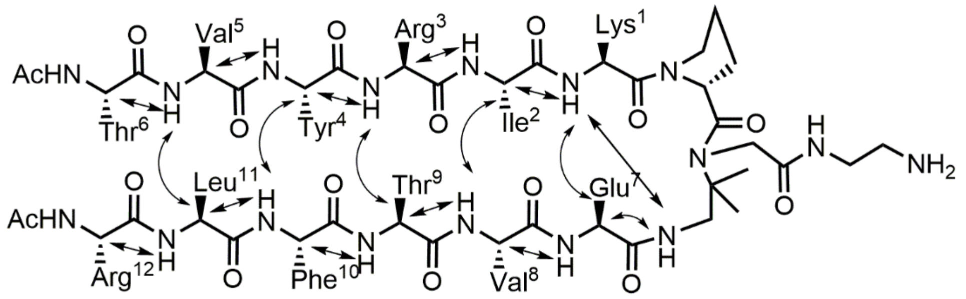

Detectable NOE contacts indicative of parallel beta-sheet folding

Residue	H-atom	Residue	H-atom	NOE intensity
**Val**^{5}	HN	**Thr**^{6}	HA	Strong
**Val**^{5}	HN	**Leu**^{11}	HA	Week
**Tyr**^{4}	HN	**Val**^{5}	HA	Medium
**Arg**^{3}	HN	**Tyr**^{4}	HA	Strong
**Arg**^{3}	HN	**Thr**^{9}	HA	Medium
**Ile**^{2}	HN	**Arg**^{3}	HA	Medium
**Lys**^{1}	HN	**Ile**^{2}	HA	Strong
**Lys**^{1}	HN	**Glu**^{7}	HA	Strong
**Lys**^{1}	HN	**DADME**	HN2	Medium
**DADME**	HN	**Glu**^{7}	HA	Strong
**Glu**^{7}	HN	**Val**^{8}	HA	Medium
**Val**^{8}	HN	**Thr**^{9}	HA	Strong
**Val**^{8}	HN	**Ile**^{2}	HA	Strong
**Thr**^{9}	HN	**Phe**^{10}	HA	Medium
**Phe**^{10}	HN	**Leu**^{11}	HA	Strong
**Phe**^{10}	HN	**Tyr**^{4}	HA	Strong
**Leu**^{11}	HN	**Arg**^{12}	HA	Strong

Chemical shift deviation

Chemical shift deviation of the amino acid α-hydrogen with respect to the random coil state (CDS, $\Delta\delta C_{\alpha}H$ = δCαH (observed) – δCαH (random coil), were determined for peptide **7a** and **8**, using the random coil data from the original report by Richard and co-workers.[10]

Table 3. Comparison of DSC of peptide **7a** and **8**.

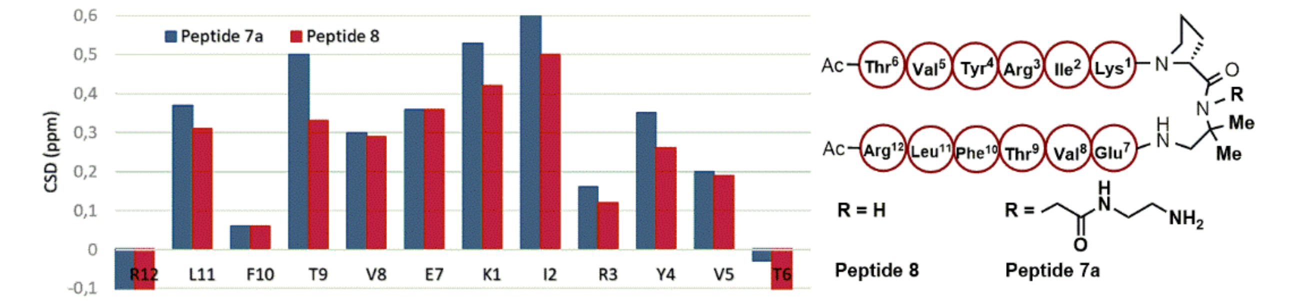

	Lys[1]	Ile[2]	Arg[3]	Tyr[4]	Val[5]	Thr[6]	Glu[7]	Val[8]	Thr[9]	Phe[10]	Leu[11]	Arg[12]
$\delta C_{\alpha}H$ Random	4.36	3.95	4.38	4.60	3.95	4.35	4.29	3.95	4.35	4.66	4.17	4.38
δHCα Peptide **7a**	**4.89**	**4.55**	**4.54**	**4.95**	**4.15**	**4.32**	**4.65**	**4.25**	**4.85**	**4.72**	**4.54**	**4.24**
$\Delta\delta C_{\alpha}H$ Peptide **7a**	**0.53**	**0.60**	**0.16**	**0.35**	**0.20**	**-0.03**	**0.36**	**0.30**	**0.50**	**0.06**	**0.37**	**-0.14**
$\delta C_{\alpha}H$ Peptide **8**	**4.78**	**4.45**	**4.50**	**4.86**	**4.14**	**4.24**	**4.65**	**4.24**	**4.68**	**4.72**	**4.48**	**4.27**
$\Delta\delta C_{\alpha}H$ Peptide **8**	**0.42**	**0.50**	**0.12**	**0.26**	**0.19**	**-0.11**	**0.36**	**0.29**	**0.33**	**0.06**	**0.31**	**-0.11**

[10] D. S. Wishart, B. D. Sykes, F. M. Richards, *Biochemistry*, **1992**, *31*, 1647.

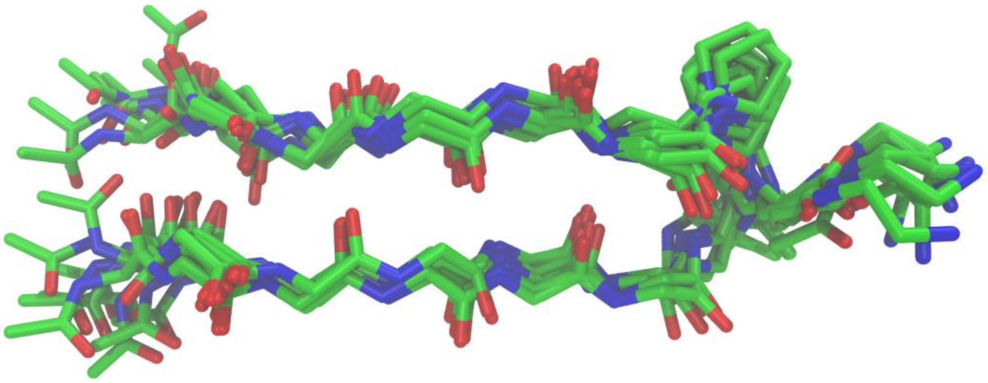

Figure 49. Superposition of the 10 lowest-energy conformations of peptide **7a** in aqueous solution.

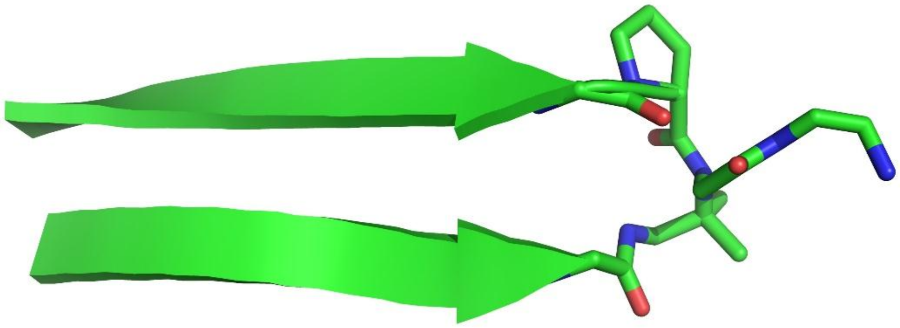

Figure 50. Most favorable conformation of peptide **7a** in aqueous solution.

Aggregation study of peptide 7a by ^{1}H-NMR

Three samples of peptide **7a**, were prepared at 0.5 mM, 1 mM and 2.5 mM concentrations in 9:1 H_2O:D_2O, pH 3.8, 100 mM sodium deuterioacetate buffer and analyzed by ^{1}H NMR. In all cases, sharp lines were observed and the superposition of the three spectra in the range of amide bonds (6.5 - 9 ppm) showed no change in the chemical shifts indicating that the increase of concentration from 0.5 mM to 2.5 mM does not induce aggregation. The peptide solutions were also checked for stability for six months, showing no change in the intensity of the signals or apparent precipitation, confirming that the peptide is not aggregated in solution.

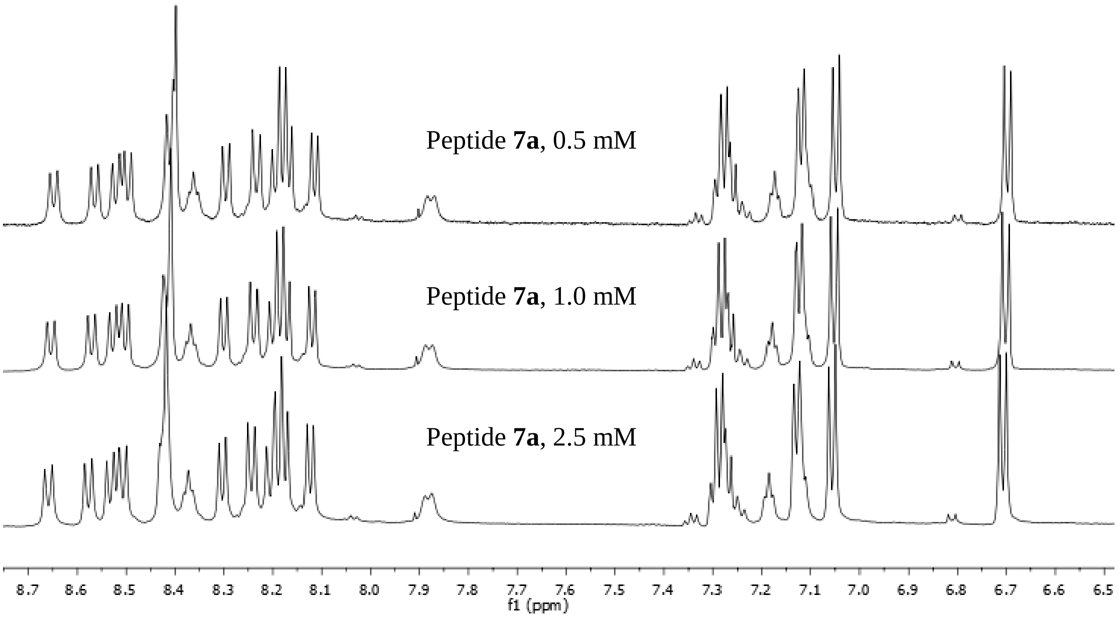

Figure S51. ^{1}H NMR spectra (600 MHz, 9:1 H_2O:D_2O, 100 mM sodium deuterioacetate buffer, pH 3.8) of peptide **7a** at 0.5 mM, 1.0 mM and 2.5 mM concentrations.
